# Inter-rater reliability of data elements from a prototype of the Paul Coverdell National Acute Stroke Registry

**DOI:** 10.1186/1471-2377-8-19

**Published:** 2008-06-11

**Authors:** Mathew J Reeves, Andrew J Mullard, Susan Wehner

**Affiliations:** 1 Department of Epidemiology, College of Human Medicine, Michigan State University, East Lansing, MI, USA

## Abstract

**Background:**

The Paul Coverdell National Acute Stroke Registry (PCNASR) is a U.S. based national registry designed to monitor and improve the quality of acute stroke care delivered by hospitals. The registry monitors care through specific performance measures, the accuracy of which depends in part on the reliability of the individual data elements used to construct them. This study describes the inter-rater reliability of data elements collected in Michigan's state-based prototype of the PCNASR.

**Methods:**

Over a 6-month period, 15 hospitals participating in the Michigan PCNASR prototype submitted data on 2566 acute stroke admissions. Trained hospital staff prospectively identified acute stroke admissions, abstracted chart information, and submitted data to the registry. At each hospital 8 randomly selected cases were re-abstracted by an experienced research nurse. Inter-rater reliability was estimated by the kappa statistic for nominal variables, and intraclass correlation coefficient (ICC) for ordinal and continuous variables. Factors that can negatively impact the kappa statistic (i.e., trait prevalence and rater bias) were also evaluated.

**Results:**

A total of 104 charts were available for re-abstraction. Excellent reliability (kappa or ICC > 0.75) was observed for many registry variables including age, gender, black race, hemorrhagic stroke, discharge medications, and modified Rankin Score. Agreement was at least moderate (i.e., 0.75 > kappa ≥; 0.40) for ischemic stroke, TIA, white race, non-ambulance arrival, hospital transfer and direct admit. However, several variables had poor reliability (kappa < 0.40) including stroke onset time, stroke team consultation, time of initial brain imaging, and discharge destination. There were marked systematic differences between hospital abstractors and the audit abstractor (i.e., rater bias) for many of the data elements recorded in the emergency department.

**Conclusion:**

The excellent reliability of many of the data elements supports the use of the PCNASR to monitor and improve care. However, the poor reliability for several variables, particularly time-related events in the emergency department, indicates the need for concerted efforts to improve the quality of data collection. Specific recommendations include improvements to data definitions, abstractor training, and the development of ED-based real-time data collection systems.

## Background

The Paul Coverdell National Acute Stroke Registry (PCNASR) is a multi-state network of hospital-based registries whose primary objective is to establish a system for monitoring and improving the quality of care provided to acute stroke patients [1,2]. Between 2001 and 2004 the PCNASR established prototype registries in eight states including Michigan [3]. These prototypes reported gaps between treatment guidelines and actual hospital care that highlighted the need for quality improvement initiatives [2,4,5].

Data from the PCNASR are used to construct specific performance measures that measure the quality of care [6,7]. Like all registries the value of the PCNASR depends in part upon the reliability of the data it collects, particularly those data elements that are used to define performance measures. Given that registry data are collected by multiple abstractors at different hospitals, data quality is vulnerable to several sources of error including mistakes in data interpretation and data entry [8]. The quality of registry data can be negatively influenced by several different factors, including inadequate abstractor training and monitoring, the use of ambiguous data definitions, and differences in information accessibility i.e., missing medical record data, or access to information sources outside the medical record [9,10]. To minimize such errors and enhance data reliability for the prototype registries, the PCNASR expert panel developed a set of standardized data elements and definitions [1,2]. Prototypes were also encouraged to adopt standardized methods for promoting the quality data, including training in chart abstraction, evaluation of case ascertainment, and assessment of inter-rater reliability of collected data [11,12]. A previous study reported on the results of a chart audit designed to compare the completeness and accuracy of data collected by prospective and retrospective prototypes of the PCNASR [13]. This study found few differences between prospective and retrospective prototypes, but found a high percentage of missing information associated with pre-hospital EMS data, thrombolytic treatment, and stroke signs and symptoms. However, this study did not examine the inter-rater reliability or agreement between hospital abstractors and auditors for individual data elements. The objective of our study was therefore to assess the inter-rater reliability of individual data elements in a random sample of subjects from the Michigan registry prototype.

## Methods

### Registry design

The MASCOTS (Michigan Acute Stroke Care Overview & Treatment Surveillance System) registry was one of the eight state-based prototype registries developed by the PCNASR. Detailed descriptions of the MASCOTS registry are available elsewhere [2,14]. In order to obtain a representative sample of hospitals providing acute stroke care in Michigan, the registry used a modified, stratified, single-stage cluster design [15], to obtain a sample of 16 hospitals. First, 8 hospitals were selected with certainty from 4 urban communities that were already participating in a community-based stroke project. These hospitals represented larger academic and non-academic urban institutions. Second, the remaining 114 acute care hospitals in the state that provided care to > 30 stroke cases in year 2000 were ranked according to size, and four equal sized strata created. Two hospitals were then randomly selected from each of the 4 strata, resulting in a final sample of 16 hospitals. The 16 selected hospitals were responsible for providing care to approximately 25% of stroke cases statewide, and analysis of their size, geographical location, teaching status, and county-level population demographics indicated that they were broadly representative of all hospitals in the state [14].

On-site coordinators ascertained acute stroke admissions using prospective methods. Between May and October 2002, the registry collected data on 2,566 confirmed acute stroke admissions from 15 hospitals (one hospital closed shortly after sampling). The MASCOTS project was granted an expedited status by hospital institutional review boards, because its primary purpose was quality improvement and no direct patient contact was involved.

### Data collection and quality control

The original PCNASR data elements included 63 core items and 22 optional items organized into twelve sections with definitions for each item. A copy of the original data elements (see Additional file 1: Original PCNASR data elements_2002.pdf) and the most current version [16] (see Additional file 2: Current PCNASR data elements_2007.pdf) are available on-line. Chart abstractions were performed at each hospital by either the site coordinator or a member of its quality improvement staff (we refer to the hospital staff as hospital abstractors). Prior to the start of the study, hospital abstractors attended a day long training session that covered all data collection steps including a detailed review of the data elements and definitions. Throughout the data collection period, project staff made regular site visits and provided supplemental training and technical support by telephone or e-mail. Hospital abstractors collected data using either a hand held computer (4 sites) or a paper abstraction tool (11 sites). Data were then entered and stored on secure desktop computers and transmitted to the central study office every other week. Staff at the central study office performed quality control using SAS software, Version 8.2 (SAS Institute Inc., Cary, NC, USA) to generate automated discrepancy reports to identify missing or invalid responses which were then sent back to the hospital abstractors for resolution. Only missing data attributed to being not documented in the medical record was considered valid. An abstracted record must have generated zero discrepancies before being classified as "closed."

### Audit sampling and chart re-abstraction

To evaluate the inter-rater reliability of the MASCOTS registry data, independent site audits were performed by a research nurse (SW) with extensive experience in acute stroke care and chart abstraction (we refer to this person as the audit abstractor). The audit sampling procedure randomly selected a block of eight sequential acute stroke admissions from each of the 15 hospitals' suspect stroke logbooks, resulting in 120 (8 × 15) chart re-abstractions. The number of re-abstractions was based on a combination of practical concerns (the number of charts that could be re-abstracted in a one-day site visit), as well as formal sample size calculations for the kappa statistic [17]. Using alpha and beta error rates of 0.05 and 0.2, respectively, when testing for a statistical difference between moderate (i.e., 0.40) and high (i.e., 0.75) kappa values, sample size estimates ranged from 77 to 28 when the trait prevalence was varied between 10% and 50%. Thus, the initial target sample size of 120 provided ample power to detect clinically important registry-level differences in reliability, however, this study was not powered to examine hospital-level differences.

To preserve the integrity of the audit, hospital abstractors were blinded to both the sampling procedure and the audit date. Of the 120 admissions, 3 were later determined to be non-stroke cases, and 13 did not have a chart available during the audit visit, leaving a total of 104 confirmed acute stroke admissions that underwent chart re-abstraction. This audit data was then compared to the "closed" registry data.

### Statistical analysis

Measures of inter-rater reliability were calculated for nominal variables with at least 30 records, and continuous and ordinal variables with at least 10 records. We included time variables (formatted as the number of minutes past midnight), but excluded dates and text data. For nominal variables, we configured individual response options as component dichotomies (positive or negative) [18]. For example, race was analyzed on six separate component dichotomies (white, black, Asian, American Indian, other, and not documented). Variables with the response options of 'No,' 'Unknown,' or 'Not documented' were coded as negative – an approach consistent with documentation by exception. We calculated kappa statistics and 95% lower confidence limits (95% LCL) as a measure of inter-rater reliability that adjusts for chance agreement [19]. To interpret kappa, we used criteria where a kappa ≥ 0.4 suggests moderate agreement, and a kappa ≥ 0.75 suggests excellent agreement.

Kappa works best when the positive and negative ratings are balanced. However, when ratings are nearly all positive or all negative because the data is highly unbalanced, kappa becomes highly sensitive to small departures from perfect concordance [19,20]. To aid interpretation of kappa, we therefore calculated measures of trait prevalence and rater bias [20]. To estimate the trait prevalence, we used the prevalence index, PI = (*a *- *d*)/*N*, where *a *and *d *are concordant ratings between the two raters and *N *is the sum of all ratings [21]. The PI can take values from -1 to +1; it is zero when concordant ratings are balanced, -1 when a trait is always absent, and +1 when always present. In this study, we used a PI < -0.90 or > 0.90 as criteria to indicate that the data was unsuitable for the calculation of kappa.

Rater bias occurs when there is a systematic difference between raters in their tendency to make a particular rating. We estimated the extent to which the hospital abstractors systematically differed from the audit rater using the bias index, BI = (*b *- *c*)/*N*, where *b *and *c *are discordant ratings [21]. The BI can take values from -1 to +1, and is zero when there is no bias. In this study, a positive BI indicates a bias toward the hospital abstractors, and a negative BI indicates a bias towards the audit abstractor. A BI < -0.10 or > 0.10 was used as criteria to indicate a substantial bias effect.

To examine the inter-rater reliability of continuous and ordinal variables, such as age or the modified Rankin Scale (mRS), we calculated intraclass correlations (ICC) using a 2-way, random effects ANOVA model [22]. The interpretation of an ICC is similar to kappa. To show the magnitude of disagreement and to test for bias, we calculated the mean and the standard deviation of the difference between the prototype and audit raters, and used the T-test to determine statistical significance between means [23].

## Results

The demographic profile of the 104 study subjects was almost identical to the overall MASCOTS registry of 2566 acute stroke admissions [2]. Based on the data collected by the hospital abstractors, the mean age of the 104 audit subjects was 70.9 years, 59% were female, and 77% were white, 17% black, 1% other race, and 5% not documented. The distribution of stroke sub-types were 58% ischemic stroke, 8% intracranial hemorrhage (ICH), 6% subarachnoid hemorrhage (SAH), 17% TIA, and 11% stroke of uncertain type.

In this article, we report measures of agreement for key demographic variables and those data elements that are directly relevant to the calculation of the current set of stroke performance measures [6,7]. Items with either a very high or low prevalence index (i.e., PI < -0.90 or > 0.90) or those that did not meet the minimal sample size requirements are not presented (results for the full set of data elements are available on-line (see Additional file 3: Inter-rater reliability_PCNASR data elements_all.pdf). Table 1 shows the inter-rater reliability kappa estimates for nominal data related to stroke sub-type, gender, race, health insurance and arrival mode. Agreement was excellent (i.e., kappa ≥ 0.75) for hemorrhagic stroke sub-types (i.e., ICH and SAH), gender, black race, Medicare insurance, and ambulance arrival. Agreement was at least moderate (kappa ≥ 0.40) for ischemic stroke, TIA, white race, Medicaid or self-pay, non-ambulance arrival, hospital transfer and direct admit. Using the criteria of a bias index (BI) of < -0.10 or > 0.10 to indicate a substantial bias effect, there was a substantial systematic differences between hospital abstractors and the audit abstractor for the recording of white race, and race not documented (Table 1). The positive BI for white race (BI = 0.13) indicates that the hospital abstractors were more likely to mark this option compared to the audit rater, while the negative BI for race not documented (BI = -0.11) illustrates the concomitant tendency for the audit rater to record race as not documented. As expected, both of these items were associated with reduced kappa values. Table 2 presents the intraclass correlations (ICC) and measures of bias for selected continuous and ordinal variables. All variables, including age were recorded with very high reliability (ICC ≥ 0.80).

**Table 1:** Inter-rater reliability for stroke sub-type, demographic, clinical features, and pre-hospital data.

**Variable (Item Number)**	**Dichotomous response option**	***N***	PI*	**Kappa (95% LCL)†**	**BI‡**
Coverdell stroke sub-type (1.0)	Ischemic Stroke	104	0.24	0.61 (0.46)	-0.07
	Intracranial hemorrhage (ICH)	104	-0.86	0.93 (0.79)	0.01
	Subarachnoid hemorrhage (SAH)	104	-0.89	0.90 (0.72)	0.01
	Transient ischemic attack (TIA)	104	-0.64	0.70 (0.52)	-0.01
Gender (1.2)	Female	104	0.16	0.98 (0.94)	0.01
Race (1.3)	White	104	0.40	0.55 (0.38)	0.13
	Black or African American	104	-0.66	0.97 (0.90)	0.01
	Not documented (missing)	104	-0.76	0.07 (-0.13)	-0.11
Health Insurance status (1.7)	Medicare	104	0.43	0.83 (0.72)	0.03
	Medicaid	104	-0.86	0.50 (0.18)	- 0.03
	Self-pay	104	-0.89	0.52 (0.15)	0.01
Arrival mode (2.2)	Ambulance	104	-0.40	0.82 (0.69)	0.00
	Other	104	-0.14	0.59 (0.43)	-0.01
	Hospital transfer	104	-0.74	0.70 (0.50)	0.03
	Not documented (missing)	104	-0.72	0.40 (0.15)	-0.01
Direct admit (2.2b)	Yes	98	-0.88	0.47 (0.17)	0.04

* Prevalence Index (PI) is a measure of the true prevalence of the trait. PI is 0 when concordant responses are equally balanced between the 2 raters. A large negative PI indicates trait is rarely found, while a large positive PI indicates trait is common. Variables with extreme distributions i.e., PI < -0.90 or > 0.90 are not shown. **† **95% LCL = 95% Lower Confidence Limit.

**‡ **Bias Index (BI) is a measure of the relative bias between the hospital and audit abstractors. BI is 0 when there is no bias. Positive BI values indicate a bias toward the hospital abstractor, negative BI values indicates a bias towards the audit abstracter. BI values > 0.10 or < -0.10 indicate substantial bias.

**Table 2:** Intraclass correlations (ICC) and measures of bias for selected continuous and ordinal variables.

				**Difference between ratings**†		
				
**Variable (units) (Item Number)**	N*	**ICC**	**(95% LCL)**	**Mean**	**SD**‡	***P***
Age (years) (1.1)	104	0.98	(0.96)	-0.11	3.19	0.957
Stroke onset time (mins) (6.2) §	19	0.99	(0.99)	-10.00	29.72	0.903
ED arrival time (mins) (4.1)	78	0.87	(0.82)	16.55	168.07	0.775
Time first seen by doctor (mins) (4.2)	49	0.88	(0.82)	22.14	160.26	0.770
Initial brain imaging time (mins) (5.2)	30	0.93	(0.89)	-19.97	111.67	0.809
Total Cholesterol (mg/dL) (12.9)	35	0.91	(0.86)	4.29	22.03	0.705
HDL (mg/dL) (12.9)	33	0.99	(0.99)	0.33	1.34	0.916
LDL (mg/dL) (12.9)	28	1.00		0.00	0.00	1.000
Modified Rankin at discharge (Score) (12.6)	99	0.80	(0.73)	-0.01	1.10	0.968

*Where both prototype and audit data have values. †Calculated as audit abstractor minus hospital abstractor. ‡Standard deviation of the difference. §Where both auditor and prototype raters recorded onset time as specific. 95% LCL = 95% Lower Confidence Limit. P = T-test probability.

Table 3 shows the estimates of inter-rater reliability for nominal data related to the emergency department (ED) processes relevant to the decision to treat a patient with tissue plasminogen activator (tPA). These data include the documentation (i.e., presence or absence) of critical time points such as stroke onset time, ED arrival time, time first seen by a doctor, time of stroke team consult, and time of initial brain imaging. Only 2 items had excellent agreement (i.e., kappa ≥ 0.75): the presence of hemorrhage on initial brain image, and whether time (i.e., > 3 hours) was the reason that tPA was not given (Table 3). Several important variables had poor agreement (kappa < 0.40) including whether a stroke team was consulted, whether the time of the stroke team consultation was documented, and whether the time of initial brain imaging was documented. Data items relevant to understanding the onset of stroke symptoms showed only moderate reliability at best; whether the onset of symptoms was specific (i.e., known precisely) or estimated (i.e., identified as occurring within a 6 hour window) showed moderate (kappa 0.51) and poor (kappa 0.22) reliability, respectively. Finally, the source of the onset time information (i.e., witnessed, patient self report or not documented) were all unreliable measures (kappa < 0.40). Most of the items shown in Table 3 had BIs that exceeded ± 0.10 indicating substantial systematic differences between hospital abstractors and the audit rater. For example, documentation that the date and time of stroke team consult was missing (non-documented) had a strong positive bias (BI = 0.40), indicating the tendency for the hospital abstractors to record this item as missing, while the audit abstractor tended to record a time for this item. Another example of strong rater bias was whether the source of information on stroke onset time was documented or not. This item was associated with a large negative bias (BI = -0.74) indicating that the audit abstractor had a much stronger tendency to record this item as missing, as compared to the hospital abstractors (Table 3). It is important to note that when both hospital and audit abstractors did record a date and time for the ED-based processes, the results were very reliable as indicated by the high ICC results (i.e., 0.87 – 0.99) for stroke onset time, ED arrival time, time first seen by a doctor, and time of initial brain imaging (Table 2).

**Table 3:** Inter-rater reliability for Emergency Department (ED) evaluation, imaging, stroke onset time, and t-PA related data.

**Variable (Item Number)**	**Dichotomous response option**	***N***	PI*	**Kappa (95% LCL)†**	**BI‡**
Date & time ED arrival (4.1)	Not documented (missing)	90	0.63	0.59 (0.40)	0.07
Date & time first seen by doctor (4.2)	Not documented (missing)	90	0.21	0.44 (0.26)	0.04
Stroke team consult (4.3)	Yes	90	-0.12	0.30 (0.10)	0.01
Date & time stroke team consult (4.1)	Not documented (missing)	40	0.20	0.02 (-0.20)	0.40
Stroke/TIA diagnosed in ED (4.6)	Yes	90	0.86	0.26 (-0.08)	0.03
Type of initial brain image (5.1)	CT	104	0.89	0.71 (0.41)	-0.03
Date & time initial brain image (5.2)	Not documented (missing)	104	-0.08	0.32 (0.15)	0.17
Date & time image results first known (5.5)	Not documented (missing)	103	-0.46	0.25 (0.10)	0.29
Intracranial hemorrhage on image (5.6)	Yes	103	-0.71	0.84 (0.70)	0.00
Stroke onset time documented (6.1)	Yes – Specific time	104	-0.40	0.51 (0.33)	0.13
	Yes – Estimated time	104	-0.17	0.22 (0.06)	-0.25
	No	104	-0.44	0.48 (0.30)	0.12
Source of onset time data if specific (6.2b)	Witnessed	38	-0.37	0.21 (-0.05)	0.26
	Patient self-report	38	-0.45	0.06 (-0.11)	0.39
	Not documented (missing)	38	-0.26	0.00	-0.74
Reasons for non-treatment with tPA (8.1)	Time	86	-0.83	0.78 (0.54)	0.03
	Not documented (missing)	86	0.59	0.54 (0.33)	-0.10

* PI = Prevalence Index (see Table 1 footnote for interpretation).

† 95% LCL = 95% Lower Confidence Limit.

‡ BI = Bias Index (see Table 1 footnote for interpretation).

ED = Emergency department, CT = computer tomography, tPA = tissue plasminogen activator (thrombolysis treatment)

The inter-rater reliability of data related to medical history, and in-hospital diagnostic procedures and treatments are shown in Table 4. Most of the medical history items were recorded with good to excellent agreement (i.e., kappa values > 0.70), while stroke/TIA, coronary heart disease (CAD), and smoking were moderately reliable (i.e., kappa values 0.59–0.62). In-hospital procedures such as the identification of atrial fibrillation (AF) in the hospital, anti-thrombotic treatment within 24 hours of admission, and screening for dysphagia were also moderately reliable (i.e., kappa 0.55 – 0.64). Only DVT prophylaxis showed a substantial bias effect (BI = 0.11) indicating the tendency for the hospital abstractor to more frequently record this intervention as having occurred, compared to the audit rater. Blood lipid measures i.e., total cholesterol, LDL-cholesterol showed excellent reliability and no evidence of statistically significant bias (Table 2).

**Table 4:** Inter-rater reliability for medical history, and select in-hospital diagnostic procedures, and treatments.

**Variable (Item Number)**	**Dichotomous response option**	***N***	PI*	**Kappa (95% LCL)†**	**BI‡**
Past Medical History (9.1)	Stroke/TIA	104	-0.23	0.59 (0.44)	-0.04
	Myocardial infarction	104	-0.71	0.77 (0.59)	0.02
	CAD	104	-0.26	0.61 (0.45)	-0.05
	AF	104	-0.64	0.70 (0.52)	0.01
	Hypertension	104	0.38	0.73 (0.59)	0.04
	Dyslipidemia	104	-0.40	0.72 (0.58)	0.04
	Diabetes	104	-0.48	0.80 (0.67)	0.04
	Smoking	104	-0.59	0.62 (0.43)	0.03
AF present in hospital (10.1)	Yes	104	0.63	0.59 (0.40)	0.07
Anti-thrombotic therapy initiated (10.4)	Within 24 hours	104	0.53	0.55 (0.37)	0.09
DVT prophylaxis initiated (10.6)	Within 48 hours	104	-0.49	0.43 (0.24)	0.11
Screening for dysphagia (10.7)	Yes	104	-0.32	0.64 (0.48)	0.05

* PI = Prevalence Index (see Table 1 footnote for interpretation). † 95% LCL = 95% Lower Confidence Limit. ‡ BI = Bias Index (see Table 1 footnote for interpretation). TIA = transient ischemic attack, CAD = coronary heart disease, AF = atrial fibrillation, DVT = deep vein thrombosis.

Medications used at discharge were recorded with excellent agreement (Kappa > 0.75) (Table 5), however, discharge destination, specifically whether discharge home or to a skilled nursing facility both showed surprisingly poor reliability (i.e., kappa < 0.4) and high positive bias (BI > 0.10) indicating the tendency for the hospital abstractors to record these events as occurring compared to the audit rater. Ambulatory status at discharge was moderately reliable when recorded as either able or unable to ambulate, but the middle category (subject requires assistance to ambulate) showed poor reliability (kappa 0.19). The modified Rankin score (MRS) showed excellent agreement (ICC 0.80) and no evidence for statistically significant rater bias (Table 2).

**Table 5:** Inter-rater reliability for discharge related data.

**Variable (Item Number)**	**Dichotomous response option**	***N***	PI*	**Kappa (95% LCL)†**	**BI‡**
Discharge destination (12.3)	Home (routine)	104	-0.22	0.38 (0.23)	0.26
	Skilled nursing facility	104	-0.88	0.00	0.13
	Rehabilitation	104	-0.78	0.30 (0.23)	0.14
Ambulatory status at discharge (12.5)	Yes – Independent	104	0.19	0.52 (0.36)	0.04
	Yes – With assistance	104	-0.64	0.19 (-0.03)	-0.09
	No – Unable	104	-0.71	0.69 (0.49)	0.02
On lipid lowering medication at D/C (12.11)	Yes	104	-0.35	0.78 (0.65)	0.04
On diabetes medication at D/C (12.13)	Yes	104	-0.55	0.86 (0.75)	0.03
On anti-hypertensive medication at D/C (12.16)	Yes	104	-0.44	0.78 (0.59)	-0.02
On anti-thrombotic therapy at D/C (12.20)	Yes – Aspirin	104	-0.17	0.76 (0.64)	-0.02
	Yes – Warfarin	104	-0.58	0.94 (0.86)	0.00
	None given	104	-0.60	0.76 (0.60)	-0.02

* PI = Prevalence Index (see Table 1 footnote for interpretation). † 95% LCL = 95% Lower Confidence Limit. ‡ BI = Bias Index (see Table 1 footnote for interpretation). D/C = discharge, skilled nursing facility = nursing home.

## Discussion

The use of performance measures to measure the quality of patient care is the primary mechanism by which improvements to the quality of acute stroke care can be made on a system wide basis [1,2,24]. However, for this approach to be successful it is vital that the data from which performance measures are constructed be reliable. We examined the inter-rater reliability of individual data elements in the PCNASR, and found that while most of them had moderate to excellent inter-rater reliability, some measures, especially ED-based time variables (i.e., time of stroke onset, stroke team consultation and brain imaging), showed poor reliability. Time related events are particularly important for stroke care because available acute therapy, for example intravenous tPA, requires administration within 3 hours of symptom onset [25]. The most common problem affecting these data elements was the large amount of missing data; data was recorded by both abstractors in only a minority of subjects. For example, a specific stroke onset time was recorded by both the hospital abstractor and audit abstractor in only 18% (19/104) of subjects, while brain imaging time was recorded by both abstractors in only 29% (30/104) of subjects. Similar to our findings, the previous study [13] that examined data quality in the context of the PCNASR also found that time-related events, particularly those associated with pre-hospital and ED environments, were the most likely to be missing.

Another problem area was the recording of the onset time of stroke symptoms which is another critical data point in determining eligibility for acute thrombolytic therapy. In the PCNASR information on stroke onset time is recorded in terms of whether the onset of symptoms is known to be specific (i.e., known precisely), or estimated (i.e., known to have occurred within a 6 hour window), or unknown. This data element had significant problems with both low reliability and substantial bias, particularly for the 'estimated time' response option. Similarly, the recording of the source of the stroke onset time data – whether the information was provided by a witness, by self report, or was not documented was also problematic in terms of poor reliability and bias. We have previously reported wide variability in the documentation of onset time between PCNASR prototypes, which implies problems with the recording of this information in the charts [2]. Other studies examining stroke onset time data have found a similar high degree of missingness or inaccurate data, particularly when the data is abstracted retrospectively from medical charts [26,27]. The study by Evenson *et al *[26] found that information on the time of symptom onset was more complete if collected by face-to-face interview (95%) than by medical record abstraction (60%),. Given the central importance of recording stroke onset time accurately, our findings suggest the need to modify the original definitions so that they are more precise and unambiguous. An approach to the documentation of stroke onset time that addresses these issues has been recently proposed by Rosamond [28], who recommends the development of a real-time data collection system to record symptom onset time data for all patients evaluated with stroke-like symptoms in the ED. The accuracy of onset time data can also be improved by the use of standardized definitions, in particular, the adoption of a fifteen minute window for the designation of a specific onset time [28]. Subsequent revisions to the PCNASR data elements have attempted to clarify the recording of stroke onset time information [16] (see Additional file 2: Current PCNASR data elements_2007.pdf), however the instructions remain inherently complex and the reliability of this critically important data item remains in question.

Poor reliability of medical chart data can be attributed to a combination of factors including ambiguous or complicated data definitions, inadequate training, and/or problems accessing information from the charts [29-31]. The use of bias index (BI) in this study helps identify systematic differences between the hospital abstractors and the audit rater, which can imply either differences in the interpretation of the data elements or differences in access to specific information. Ambiguous or complicated data definitions likely contributed to the poor reliability and rater bias associated with two variables; date and time of stroke team consultation and DVT prophylaxis. Discussions which followed the completion of this study found that there was wide variability between hospital abstractors in their understanding of the definition of an acute stroke team; in the PCNASR a stroke team is broadly defined to include any medical personnel (i.e., physician, nurse, physician assistant) who evaluated the patient for thrombolytic (tPA) therapy in the ED. Due in part to the poor reliability of this data element, stroke team consultation is no longer included in the current registry data elements. The poor reliability and bias associated with DVT prophylaxis stemmed from the difficulty in determining who was eligible for this measure; patients who are ambulating by the second hospital day are not eligible for this intervention. However, at the time of this study, instructions on how to define ambulatory status for hospitalized patients were lacking. The current version of the registry data elements now includes a separate variable to document ambulatory status (defined as 'walking 50 feet independently or out of bed to bathroom without supervision') [16] (see Additional file 2: Current PCNASR data elements_2007.pdf).

Some of our results suggest that the reliability of certain data elements was impacted by the fact that hospital abstractors had access to information that was not available to the audit abstractor. For example, the observed bias for hospital abstractors to have rated race as white (BI = 0.13), while the audit rater tended to use race 'Not documented' (BI = -0.11), suggests that hospital abstractors were more likely to assign race based on bedside observation. Gomez *et al*[32] reported that in a sample of 70 California hospitals approximately one-half obtained data on race by observing a patient's physical appearance, and this data is not consistently recorded in the medical chart. The reliability and accuracy of race data would be improved if hospitals included race as a specific item in the medical chart, and if it were ascertained by patient self-report rather than external observation.

The findings from this and other studies [13] can be used to improve the reliability of the PCNASR data elements through a combination of steps, including modifying data elements, enhancing the clarity of data definitions, and improving abstractor training. As discussed above several data elements – stroke onset time, stroke team consultation and DVT prophylaxis, have already undergone significant changes in terms of either their structure, and/or instructions [16]. Given that many of the items with poor reliability are collected in the ED environment (e.g., stroke team consult, onset time, brain imaging time), the development of ED-based systems to capture data items in real-time should be a major priority. Such systems could be as simple as the consistent documentation by ED medical staff of specific data elements included in an ED clinical pathway, or as sophisticated as an electronic data collection system that would record these data items in real-time [33].

This study has several limitations. First, we had insufficient resources to abstract an adequate number of charts to measure inter-rater reliability at the level of each hospital. Second, the fact that the data collected by the hospital abstractors was assembled through a combination of prospective (i.e., real-time) and retrospective (i.e., chart abstraction) methods, whereas the audit abstractor had to rely on only retrospective chart abstraction, likely increased the frequency of discrepancies between the two abstractors. Several instances where the reliability of specific data elements was affected by the lack of access to data for the audit rater have already been highlighted. A third limitation is the fact that charts were unavailable for 13 patients (11%) of the original sample. However, these patients were randomly distributed across the 15 registry hospitals and so this is unlikely to bias the results in any significant way. Finally, while the inter-rater reliability for these data elements may vary across other hospitals and registries, the overall generalizability of these findings is likely to be considerable, particularly for those items that showed obvious problems with either ambiguous or complicated data definitions, or a high degree of missingness.

## Conclusion

The excellent inter-reliability of many of the data items included in the PCNASR, such as demographics, stroke subtype, past medical history, and discharge medications supports use of the registry data for construction of performance measures used to monitor care and guide QI initiatives. However, the poor reliability observed for some variables, particularly the documentation of event times will likely hinder the registry's ability to accurately monitor care. Our findings illustrate specific areas, such as the documentation of stroke onset time, where changes to data definitions, enhanced abstractor training, and the development of concurrent, real-time data collection systems should be strongly considered in order to improve the quality of that data used to track and monitor acute stroke care.

## Competing interests

The authors declare that they have no competing interests.

## Authors' contributions

MJR conceived, designed and coordinated the study, and was responsible for drafting and editing the manuscript. AJM participated in the design of the study, oversaw all data collection, and performed all statistical analyses. SW participated in the conduct of the study, and was responsible for all chart re-abstractions. All authors read and approved the final manuscript.

## Pre-publication history

The pre-publication history for this paper can be accessed here:



## Supplementary Material

Additional file 1 Original recommended data elements for the Paul Coverdell National Acute Stroke Registry – 2002. Full listing of original data elements for the PCNASR. Includes Item number, variable name, coding, definitions and formats. Click here for file

Additional file 2 Data Elements For Paul Coverdell National Acute Stroke Registry. Full listing of current data elements for the PCNASR as defined in 2007. Includes Item number, variable name, coding, definitions and formats. Click here for file

Additional file 3 Inter-rate reliability for selected data elements from the Paul Coverdell National Acute Stroke Registry. Inter-rater reliability results for all of the PCNASR data elements. Includes Item number, variable name, sample size, Prevalence index (PI), lower confidence interval of Kappa statistic, and Bias index (BI). Results were not generated for date or text fields, or for those variables that had insufficient sample size or when the PI was extreme (> 0.90 or < -0.90). Click here for file
