# Supplementary material for: Inter-rater reliability of data elements from a prototype of the Paul Coverdell National Acute Stroke Registry
Source: BMC Neurol. 2008 Jun 11;8:19. doi: 10.1186/1471-2377-8-19 (PMC2442121; doi:10.1186/1471-2377-8-19)
Supplement: Additional file 1 — Original recommended data elements for the Paul Coverdell National Acute Stroke Registry – 2002. Full listing of original data elements for the PCNASR. Includes Item number, variable name, coding, definitions and formats. [file 1471-2377-8-19-S1.pdf]

*Original recommended data elements for the Paul Coverdell National Acute Stroke Registry - 2002.**

Item	Status<br><Variable name>	Item and Categories	Suggestions, Notes, Definitions, Formats	Stroke Type
1		**Demographic Data**		All
1.1	Core<br><Age>	Age |__|__|__| years	Calculated from date of birth in medical record.	
1.2	Core<br><Gender>	Gender (Check only one)<br>“ Male<br>“ Female<br>“ Unknown		
1.3	Core<br><Race1><br><Race2><br><Race3><br><Race4><br><Race5><br><Race6><br><br><RaceOth>	Race (Check all that apply)<br>“ White<br>“ Black or African American<br>“ Asian or Pacific Islander<br>“ American Indian or Alaskan Native<br>“ Other ____________________<br>“ Not documented<br><br>A text field to document “Other” verbatim for variable <Race5>	Determined by the ER admissions document or the intake/face sheet/hospital admissions database. If not specified, check “Not documented.”<br><br>(Source for categories: AMEA OMB 15 Model)<br><br>See Appendix A for list of synonyms/indicators of race variables.	
1.4	Core<br><Ethnic>	Hispanic or Latino origin (Check only one)<br>“ Yes<br>“ No/Not documented	If not documented, check the box for “No/not documented”.<br><br>See Appendix B for list of synonyms/indicators of hispanic or latino origin.	
1.5	Core<br><Reside>	Place of Residence (Check only one)<br>“ Nursing home<br>“ Other	“Nursing home” includes an institutionalized setting where skilled nursing care is provided. “Other” is a non-institutionalized setting, such as a private home or supervised care/assisted living.	
1.6	OPTION<br><Zipcode>	ZIP code of residence |__|__|__|__|__|		

(continued)

Item	Status <Variable name>	Item and Categories	Suggestions, Notes, Definitions, Formats	Stroke Type
1.7	OPTION<br><HlthIns1><br><HlthIns2><br><HlthIns3><br><HlthIns4><br><HlthIns5><br><HlthIns6><br><HlthIns7><br><HlthIns8>	Health insurance status (Check all that apply)<br>Medicare<br>Medicaid<br>Blue Cross/Blue Shield<br>HMO/PPO<br>Other private/commercial<br>Other<br>Self Pay<br>Not documented		
2		**Pre-Hospital/Emergency Medical System (EMS) Data**		All
2.1	OPTION<br><InHospYN>	Did the stroke meeting the case definition occur in-hospital? (Check only one)<br>“ Yes → Skip to Section 5 (Imaging)<br>“ No		
2.2	Core<br><EMSMode>	Arrival Mode (Check only one)<br>“ Ambulance<br>“ Air<br>“ Ambulance hospital transfer<br>“ Other<br>“ Not documented	“Other” includes private transportation (e.g. cab, bus, car, walk-in, etc.).	
2.2b	OPTION<br><DirAdmYN>	Was the patient a direct admit?<br>“ Yes<br>“ No	Recommend collecting this item separately from item 2.2.	
2.3	Core<br><EMSShtYN>	If transport by EMS, is EMS “trip sheet” available in the medical records? (Check only one)<br>“ Yes<br>“ No → Skip to Section 4 (ED Triage)	“Trip sheet” includes any documentation from EMS for their activity. EMS information for this section may be abstracted from either an EMS form and/or the medical chart.	
2.4	Core<br><EMSRecD><br><EMSRecT><br><EMSRecND>	2. Date & time call received by EMS [Date/Time]<br>“ Not documented	mm/dd/yyyy; 24-hour clock, Check “Not documented” box to indicate that either date or time is not documented.	
2.4b	OPTION<br><EMSRecS>	a2. EMS call was assigned a stroke nature code or a priority one dispatch<br>“ Yes<br>“ No		

(continued)

Item	Status <Variable name>	Item and Categories	Suggestions, Notes, Definitions, Formats	Stroke Type
2.5	Core<br><EMSDispD><br><EMSDispT><br><EMSDispN>	b. Date & time of EMS dispatch<br>[Date/Time]<br>“ Not documented	mm/dd/yyyy; 24-hour clock, Check “Not documented” box to indicate that either date or time is not documented.	
2.6	Core<br><EMSArrD><br><EMSArrT><br><EMSArrND>	c. Date & time of EMS first patient contact<br>[Date/Time]<br>“ Not documented	mm/dd/yyyy; 24-hour clock, Check “Not documented” box to indicate that either date or time is not documented.<br>Determined by time of first contact noted on trip sheet. If not available, determined by time of first vital signs.	
2.7	Core<br><EMSDepD><br><EMSDepT><br><EMSDepND>	d. Date & time of EMS scene departure<br>[Date/Time]<br>“ Not documented	mm/dd/yyyy; 24-hour clock, Check “Not documented” box to indicate that either date or time is not documented.	
2.8	OPTION<br><EMSSyst><br><EMSDias>	Blood Pressure<br>e1. – Systolic BP |__|__|__| mmHg<br>e2. – Diastolic BP |__|__|__| mmHg		
2.8b.	OPTION<br><EMSGluc>	Blood Glucose Level	Measurement documented by EMS personnel	
2.9	OPTION<br><EMSRate>	f. Heart Rate<br>|__|__|__| beats per 20 seconds		
2.10	Core<br><EMSScaYN>	g. Was a stroke scale recorded? (Check only one)<br>“ Yes<br>“ No	Check the “yes” box if any pre-hospital stroke scale was recorded. Do not consider the Glasgow Coma Scale (GCS) for this item.	
2.11	Core<br><EMSGCSYN>	h. Was a pre-hospital Glasgow Coma Scale (GCS) recorded? (Check only one)<br>“ Yes<br>“ No	Check the “yes” box if a pre-hospital Glasgow Coma Scales was recorded, that is, a GCS administered by EMS personnel. Do not consider any GCS administered in the ED for this item.	
3		**Arrival at Emergency Department (ED) Data**		
3.1	OPTION<br><SPlace><br><br><br><br><SplaceOt>	Where did stroke occur? (Check only one)<br>“ Work<br>“ Home<br>“ Other, Specify_________________<br><br>A text field to document “Other” verbatim for variable <Splace>		All

(continued)

Item	Status <Variable name>	Item and Categories	Suggestions, Notes, Definitions, Formats	Stroke Type
4		**Emergency Department (ED)**		All
4.1	Core<br><EDTriagD><br><EDTriagT><br><EDTriagN>	Date & time of arrival in ED<br><br>[Date/Time]<br>❝ Not documented	mm/dd/yyyy; 24-hour clock, Check "Not documented" box to indicate that either date or time is not documented.<br>Determined by earliest documented time of patient arrival in ED record, which may be the time of triage or ED check-in.	
4.2	Core<br><EDMDD><br><EDMDT><br><EDMDND>	Date & time first seen by emergency department medical doctor<br><br>[Date/Time]<br>❝ Not documented	mm/dd/yyyy; 24-hour clock, Check "Not documented" box to indicate that either date or time is not documented.<br>ED MD is the first physician who sees the patient.	
4.3	Core<br><SteamYN>	Evidence in record of consultation or discussion with acute stroke team?<br>(Check only one)<br><br>❝ Yes<br>❝ No → Skip to Item 4.5	Definition of acute stroke team: Includes neurologist, neurosurgeon, interventional radiologist, or other designated physician, specifically including any MD, Physicians' Assistant (PA), or licensed Registered Nurse (RN), who documents being part of the "stroke team" or documents evaluating patient for possible "thrombolytic or TPA" therapy.	
4.4	Core<br><SteamD><br><SteamT><br><SteamND>	If yes, date & time of acute stroke consultation<br><br>[Date/Time]<br>❝ Not documented	mm/dd/yyyy; 24-hour clock, Check "Not documented" box to indicate that either date or time is not documented.<br>Documented in nurse's record or acute stroke consultant record. Includes documentation of telephone consultations.	
4.5	Core<br><EDEvalYN>	Was stroke or stroke-like symptoms documented in the emergency department evaluation? (Check only one)<br>❝ Yes<br>❝ No	As documented by either MD or RN.	
4.6	Core<br><EDDiagYN>	Was stroke/TIA one of the documented ED diagnoses? (Check only one)<br>❝ Yes<br>❝ No	As documented by either MD or RN.	

(continued)

Item	Status <Variable name>	Item and Categories	Suggestions, Notes, Definitions, Formats	Stroke Type
5		**Imaging**		All
5.1	Core<br><ImageTyp>	Type of <u>initial</u> brain image (Check only one.)<br>❝ CT<br>❝ MRI<br>❝ Not done<br>❝ Not documented	Use earliest brain image, including an image from an outside transfer hospital or the receiving hospital. Check "Not done" box when there is explicit documentation that the image was not done for a particular reason. Consider a check box for "Other" response options.	
5.2	Core<br><br><ImageD><br><ImageT><br><ImageND><br><ImageOut>	Date & time of initial brain imaging (not time dictated)<br><br>[Date/Time] → If date & time are documented, skip to Item 5.5<br><br>❝ Not documented → complete Items 5.3 & 5.4<br>❝ Outside brain imaging prior to transfer → skip to Item 5.5	mm/dd/yyyy; 24-hour clock, Check "Not documented" box to indicate that either date or time is not documented.<br>Determined by date/time recorded on the image itself <u>or</u> the date/time that the scan was performed as noted in the dictated report. Time of dictation is <u>not</u> recorded by the abstractor.<br>Check "Outside brain imaging…" box if imaging was done outside of the hospital, prior to patient transfer.	
5.3	Core<br><br><LeftEDD><br><LeftEDT><br><LeftEDND>	Date & time patient left the ED to radiology for initial image.<br><br>[Date/Time]<br>❝ Not documented	mm/dd/yyyy; 24-hour clock, Check "Not documented" box to indicate that either date or time is not documented.<br>Use MD/RN notes as source. Make sure that the date and time are not when the image was read.	
5.4	Core<br><br><RtrnEDD><br><RtrnEDT><br><RtrnEDND>	Date & time patient returned from radiology following initial imaging to the ED or returned to other location (e.g., med/surg floor, angiography suite--whichever is earlier).<br><br>[Date/Time]<br>❝ Not documented	mm/dd/yyy; 24-hour clock, Check "Not documented" box to indicate that either date or time is not documented.	
5.5	Core<br><br><KnownD><br><KnownT><br><KnownND>	Date & time of earliest documentation of imaging results known to the treating physician.<br><br>[Date/Time]<br>❝ Not documented	mm/dd/yyyy; 24-hour clock, Check "Not documented" box to indicate that either date or time is not documented.	

(continued)

Item	Status <Variable name>	Item and Categories	Suggestions, Notes, Definitions, Formats	Stroke Type
5.6	Core<br><EvHemYN>	Evidence of intracranial hemorrhage on initial image?<br>(Check only one)<br>“ Yes<br>“ No<br>“ Not documented	This would include mention of “hemorrhage” or “bleeding” in radiology report.	
6		***Signs and Symptoms Onset***		All
6.1	Core<br><OnsetSS>	Is a specific date and time of onset of acute stroke S/S documented or is an estimated time accurate to within a six-hour window available?<br>“ Specific time → skip to 6.2.a / 6.2.b<br>“ Estimated time → skip to 6.3.c / 6.3.d<br>“ No specified time → skip to 6.4.e	Date and time are considered “specific” if obtained from witnessed onset of acute stroke signs & symptoms<br><br>Definition of estimated time is accurate to within a six-hour window (i.e., morning, afternoon, evening, overnight).<br><br>Time of onset will be determined from the record using sources in the following priority:<br>1. Treating neurologist or stroke team<br>2. Treating physician (ED MD)<br>3. Treating nurse<br>4. EMS run sheet<br><br>If a time range is documented, record earlier date/time of the range.<br><br>See Appendix C for list of synonyms/indicators of stroke signs and symptoms.	
6.2	Core<br><OnsetD><br><OnsetT><br><br><OnsetSrc>	<u>IF YES – SPECIFIC TIME:</u><br>**6.2.a** Specific date and time of onset:<br>mm/dd/yyyy ___ : ___<br><br>**6.2.b** What was the source of this information?<br>“ Witnessed<br>“ Patient self-report<br>“ Not documented		

(continued)

Item	Status<br><Variable name>	Item and Categories	Suggestions, Notes, Definitions, Formats	Stroke Type
6.3	Core<br><OnEstDay><br><br><OnEstTim>	<u>IF YES – ESTIMATED TIME:</u><br>**6.3.c** Estimated date of onset: mm/dd/yyyy<br><br>**6.3.d** Estimated time of day of onset:<br>“ Morning (6am-11:59am)<br>“ Afternoon (noon-5:59pm)<br>“ Evening (6pm-11:59pm)<br>“ Overnight (midnight-5:59am)		
6.4	OPTION<br><OnEstNor>	<u>IF NO:</u><br>**6.4.e** Date last seen normal: mm/dd/yyyy		
6.5	Core<br><NIHSS><br><NIHSSND>	First NIH Stroke Scale total score recorded by hospital personnel (Enter score <u>or</u> check box)<br>___ ___<br><br>“ Not documented	Total score maximum is 2-digit.<br>First NIHSS can be recorded by either the MD or a member of the “stroke team” (including a PA or RN).	
6.6	Core<br><NIHSSD><br><NIHSSDND>	Date first NIHSS administered<br>[Date]<br>“ Not documented	mm/dd/yyyy, Check “Not documented” box to indicate that date is not documented.	
6.7	OPTION<br><NIHSS1a><br><NIHSS1b><br><NIHSS1c><br><NIHSS2><br><NIHSS3><br><NIHSS4><br><NIHSS5R><br><NIHSS5L><br><NIHSS6R><br><NIHSS6L><br><NIHSS7><br><NIHSS8><br><NIHSS9><br><NIHSS10><br><NIHSS11>	NIHSS Sub-scale Scores:<br>__ 1a. Level of Consciousness<br>__ 1b. LOC Questions<br>__ 1c. LOC Commands<br>__ 2. Best Gaze<br>__ 3. Visual<br>__ 4. Facial Palsy<br>__ 5R. Motor Arm-Right<br>__ 5L. Motor Arm-Left<br>__ 6R. Motor Leg-Right<br>__ 6L. Motor Leg-Left<br>__ 7. Limb Ataxia<br>__ 8. Sensory<br>__ 9. Best Language<br>__ 10. Dysarthria<br>__ 11. Extinction and Inattention		

(continued)

Item	Status<br><Variable name>	Item and Categories	Suggestions, Notes, Definitions, Formats	Stroke Type
7		***Thrombolytic Treatment***		Ischem only
7.1	Core<br><ThrmYN>	Did patient receive thrombolytic therapy? (Check only one)<br><br>“ Yes<br><br>“ No → Skip to Section 8 (Non-treatment with thrombolytics)	Do not include as thrombolytic therapy for indications other than ischemic stroke. That is, do not include intra-cerebral venous infusion for cerebral venous thrombosis, intraventricular infusion for intraventricular hemorrhage, intraparenchymal infusion for percutaneous aspiration of intracerebral hematoma, myocardial infarction, PE, or peripheral clot.<br><br>See Appendix D for list of synonyms of thrombolytic drugs.	
7.2	Core<br><ThrmMode>	Route of thrombolytic delivery (Check only one.)<br>“ Intravenous<br>“ Intra-arterial (via angiography)<br>“ Intravenous and intra-arterial<br>“ Not documented		
7.3	Core<br><ThrmInvs>	Investigational therapy for acute ischemic stroke? (Check only one)<br><br>“ Yes<br>“ No/Not documented	Determined by documentation of an IRB-approved study as indicated by the presence of an informed consent form or a physician note that the patient is entering a stroke study. Include studies involving thrombolytics, mechanical, or neuroprotectives.	
7.4	Core<br><br><ThrmD><br><ThrmT><br><ThrmND>	Date & time of initiation of thrombolytic therapy.<br><br>[Date/Time]<br>“ Not documented	mm/dd/yyyy; 24-hour clock, Check “Not documented” box to indicate that either date or time is not documented.<br><br>For IV therapy, date/time when nurse documents start of infusion.<br><br>For IA therapy, time noted in nurse’s, radiologist’s or other MD’s note as to when infusion of thrombolytic began. If not noted, then use time of start of angiogram case based on radiology report or MD/RN notes.	

(continued)

Item	Status<br><Variable name>	Item and Categories	Suggestions, Notes, Definitions, Formats	Stroke Type
7.5	Core<br><br><br><ThrmCmp1><br><ThrmCmp2><br><br><ThrmCmp3>	Complications of thrombolytic therapy<br>(Check all that apply among responses or not applicable.)<br><br>“ Symptomatic intracranial hemorrhage<br>“ Life threatening, serious systemic hemorrhage<br><br>“ Not applicable<br><br>If Section 7 is completed → Skip to Section 9	Definition for symptomatic hemorrhage: CT hemorrhage within 36 hours shows intracranial bleed AND physician’s notes indicate clinical deterioration due to hemorrhage.<br><br>See Appendix E for list of synonyms/indicators of intracranial hemorrhage.<br><br>Definition for systemic hemorrhage: $\geq$ 3 transfused units of blood within 7 days or discharge (which ever is earlier) AND physician note attributing bleeding problem as reason for transfusion.<br><br>Check “Not applicable” box to indicate that patient did not experience either symptomatic intracranial hemorrhage or life threatening, serious systemic hemorrhage as complications of thrombolytic therapy.	

(continued)

Item	Status<br><Variable name>	Item and Categories	Suggestions, Notes, Definitions, Formats	Stroke Type
8		**Non-Treatment with Thrombolytics**<br>Section 8 completed only if thrombolytic therapy not given or started.		Ischem only
8.1	Core<br><br><br><NonTrt01><br><NonTrt02><br><NonTrt03><br><NonTrt04><br><NonTrt05><br><NonTrt06><br><NonTrt07><br><NonTrt08><br><NonTrt09><br><NonTrt10><br><NonTrt11><br><NonTrt12><br><br><NonTrt13><br><NonTrt14><br><NonTrt15><br><NonTrt16><br><NonTrt17><br><NonTrt18><br><NonTrt19><br><NonTrt20> &<br><NonTrtOt><br><NonTrt21>	Physician documented reasons indicated for non-treatment with thrombolytics (these are not necessarily contraindications)<br>(Check all that apply.)<br>“ Time<br>“ Uncontrolled hypertension<br>“ Rapid improvement<br>“ CT findings<br>“ Stroke severity – Too mild<br>“ Stroke severity – Too severe<br>“ Seizure at onset<br>“ Recent surgery/trauma (<15 days)<br>“ Recent IC surgery (3 mo.) head trauma/stroke<br>“ Pt./Family refused<br>“ Consent not obtainable<br>“ History of intracranial hemorrhage or brain aneurysm or vascular malformation or brain tumor<br>“ Age<br>“ Active internal bleeding (<22 days)<br>“ Platelet count (<100,000)<br>“ Abnormal aPTT or PT<br>“ Glucose < 50 mg/dl or > 400 mg/dl<br>“ No IV access<br>“ Life expectancy < 1 year or severe co-morbid illness<br>“ Other: ______________________<br><br>“ Not Documented	Document under ‘Other’ if other research protocol was used.<br><br>Use the “Other” category to specify reasons for non-treatment with thrombolytics that were specifically stated in the physician’s notes. Use item 8.2 to specify reasons for non-treatment with thrombolytics that were implied, but not explicitly stated, as reasons for non-treatment.<br><br>Variable <NonTrt20> is a check box.<br>Variable <NonTrtOt> is a text field to record Other responses.	
8.2	Core<br><NonTrtCo>	Comment field for non-treatment reasons	This field is used for documenting Other verbatim <NonTrt20> as well as other notes/observations regarding non-treatment.	

(continued)

Item	Status<br><Variable name>	Item and Categories	Suggestions, Notes, Definitions, Formats	Stroke Type
9		**Medical History**		
9.1	Core<br><br><MedHist01><br><MedHist02><br><MedHist03><br><MedHist04><br><MedHist05><br><MedHist06><br><MedHist07><br><MedHist08><br><MedHist09><br><MedHist10>	Documented past medical history of any of the following:<br>(Check all that apply.)<br>“ Stroke/Transient ischemic attack/VBI<br>“ Myocardial infarction (MI)<br>“ Coronary artery disease (CAD)<br>“ Atrial fibrillation<br>“ Heart failure (CHF)<br>“ Valve prosthesis<br>“ Hypertension<br>“ Dyslipidemia<br>“ Diabetes mellitus (DM)<br>“ Smoking	Check item if documented by physician or nurse in admission or discharge notes.<br><br>Coronary Artery Disease (CAD) - Documented evidence in medical record of a specific mention of the presence of CAD or a revascularization procedure, such as coronary bypass grafting or stenting. Documentation of angina is NOT enough to be defined as CAD.<br><br>Smoking history – patient has smoked at least one cigarette within the past year.<br><br>See Appendix F for list of synonyms/indicators of past medical history variables.	All
10		**In-Hospital Diagnostic Procedures and Treatment**		
10.1	Core<br><AFibYN>	Was atrial fibrillation present in the hospital? (Check/Not Check)<br>“ Yes [Note: This response is used as a filter in item 12.22]	Atrial fibrillation includes A-flutter.	Ischem only
10.2	Core<br><br><CVDupU><br><CVMRA><br><CVCTA><br><CVCA><br><CVTD><br><CVNoDoc>	Which of the following tests were documented to evaluate the cerebrovasculature? (Check all that apply.)<br>“ Duplex ultrasound<br>“ MR Angiogram<br>“ CT Angiogram<br>“ Cerebral Angiogram<br>“ Transcranial Doppler<br>“ None documented	Documentation includes physician’s notes or formal report in the medical record.<br><br>See Appendix G for list of synonyms/indicators of cerebrovasculature tests.	All
10.3	OPTION<br><StnTrtYN>	If stenosis documented, was intervention considered?<br>(Check only one)<br>“ Yes<br>“ No/Not documented	Interventions include endarterectomy, stenting, angiograms, and medical therapy.<br><br>Definition of “considered”: discussed, planned or performed.	Ischem only

(continued)

Item	Status<br><Variable name>	Item and Categories	Suggestions, Notes, Definitions, Formats	Stroke Type
10.4	Core<br><AthrTime>	Time of initiation of any anti-thrombotic therapy after admission. (Check only one)<br>“ 0 – 24 hours<br>“ >24 hours<br>“ Not initiated<br>“ Not documented		Ischem only
10.5	Core<br><br><AthrTy01><br><AthrTy02><br><AthrTy03><br><AthrTy04><br><AthrTy05><br><AthrTy06><br><AthrTy07><br><AthrTy08><br><AthrTy09><br><AthrTy10><br><AthrTy11>	If initiated, what anti-thrombotic therapy given during acute hospital care? (Check all that apply.)<br>“ Aspirin<br>“ Aggrenox<br>“ Warfarin/Coumadin<br>“ Ticlopidine/Ticlid<br>“ Dipyridamole/Persantine<br>“ Clopidogrel/Plavix<br>“ Heparin SQ<br>“ Heparin IV<br>“ LMW Heparin<br>“ Other anti-thrombotic<br>“ Not Given	Document anti-thrombotic therapy administered during acute medical care received upon hospital admission. Do not include therapy received after transfer to an inpatient or rehabilitation unit.<br><br>See Appendix H for list of synonyms/inclusions of anti-thrombotic drugs.	Ischem only
10.6	Core<br><DVTProYN>	Was DVT prophylaxis initiated by 2nd hospital day? (Check only one)<br>“ Yes<br>“ No<br>“ Not applicable (patient ambulating or already receiving anticoagulant)<br>“ Not documented	DVT prophylaxis includes drugs/pharmacotherapy and physical mechanisms, such as vascular support hose with pneumatic compression devices.<br>If patient already receiving anti-coagulant (drugs that prevent blood clotting) prior to hospital admission, then mark the checkbox for not applicable.	All
10.7	Core<br><DysphaYN>	Screening for dysphagia? (Check/Not Check)<br>“ Yes	Documented in nurse’s notes or speech/swallowing consult. Types of dysphagia screen include simple water swallow test, formal speech evaluation, and other tests such as barium swallow or video fluoroscopy.	All
10.8	Core<br><NeuroYN>	Was a neurologist or neurosurgeon involved in the care of the patient during hospitalization? (Check/Not Check)<br>“ Yes	“Involved in the care” means there is a written record that either a neurologist or neurosurgeon evaluated the patient at any time during hospitalization.	All

(continued)

Item	Status <Variable name>	Item and Categories	Suggestions, Notes, Definitions, Formats	Stroke Type
10.9	OPTION <CovYN>	Is there an order in the medical record that limits the medical care provided, such as "Do not resuscitate " or "Comfort/supportive care only"?<br>" Yes<br>" No		
11		**Other In-Hospital Complications**		All
11.1	Core <DVTDocYN>	Was DVT documented? (Check/Not Check)<br>" Yes	Confirmed by ultrasound or venous imaging.<br>Items 11.1, 11.2, and 11.3 refer to in-hospital acquired events requiring treatment. Pre-existing conditions and therapy present prior to admission should not be counted in responding to these data elements.	
11.2	Core <PneumYN>	Clinical mention of pneumonia, and treatment with antibiotic for that problem? (Check/Not Check)<br>" Yes	Physician documentation of definite or possible.<br>Items 11.1, 11.2, and 11.3 refer to in-hospital acquired events requiring treatment. Pre-existing conditions and therapy present prior to admission should not be counted in responding to these data elements.	
11.3	Core <UTIYN>	Clinical mention of urinary tract infection (UTI), and treatment with antibiotic for that problem? (Check/Not Check)<br>" Yes	Physician documentation of definite or possible.<br>Items 11.1, 11.2, and 11.3 refer to in-hospital acquired events requiring treatment. Pre-existing conditions and therapy present prior to admission should not be counted in responding to these data elements.	
12		**Discharge Data**		
12.1	Core <DschrgD> <DschrgND>	Date of discharge from hospital<br>[Date]<br>" Not documented	mm/dd/yyyy, Check "Not documented" box to indicate that date is not documented.	All
12.2	Core <ICD9>	ICD-9 discharge diagnosis related to stroke<br><br>— — — . — —	Use XXX.YY format; allow only one ICD-9 code.<br><br>Determined by ICD-9 code recorded in chart.	All
12.2a	Core <ICD9Prin>	Was this the principal discharge ICD-9 diagnosis?<br>" Yes<br>" No		All

(continued)

Item	Status <Variable name>	Item and Categories	Suggestions, Notes, Definitions, Formats	Stroke Type
12.3	Core<br><DschDest>	Discharge destination (Check only one.)<br>“ Code 1. Discharge to home or self-care (routine discharge)<br>“ Code 2. Discharge/transfer to another short-term general hospital for inpatient care<br>“ Code 3. Discharge/transfer to skilled nursing facility (SNF)<br>“ Code 4. Discharge/transfer to intermediate care facility (ICF)<br>“ Code 5. Discharge/transfer to another type of institution for inpatient care or referred for outpatient services to another institution<br>“ Code 6. Discharge/transfer to home under care of organized home health service organization<br>“ Code 7. Left against medical advice or discontinued service<br>“ Code 8. Discharge/transfer to home under care of a home IV provider<br>“ Code 9. Admitted as inpatient to hospital<br>“ Code 20. Expired/dead<br>“ Other UB-92 codes<br>“ Disposition not documented	[Reference source: UB-92 Codes]<br><br>Code numbers correspond to UB-92 codes for discharge.<br><br>Suggest capturing 2-digit UB-92 code.	All
12.4	Core<br><RehabYN>	If 12.3 = 5, then complete Item 12.4. All others skip to 12.5.<br>Was patient discharged to a hospital-based acute rehabilitation service? (Check only one.)<br>“ Yes<br>“ No/Not documented	Include any acute rehabilitation service within same hospital or at another hospital.	All
12.5	Core<br><FunStat>	Functional status at discharge. (Check only one.)<br>“ Able to ambulate independently.<br>“ Ambulates with assistance from another individual<br>“ Not able to ambulate<br>“ Not documented	Definition of “ambulate independently”: includes assistance with cane, walker, etc., as long as the person can walk without the assistance of another human being. If Item 12.3= Code 20 Expired/dead and the patient expired during hospitalization, item 12.5 can be skipped.	All

(continued)

Item	Status <Variable name>	Item and Categories	Suggestions, Notes, Definitions, Formats	Stroke Type
12.6	OPTION<br><Rankin><br><RankinND>	Modified Rankin Scale at discharge<br>(Enter scale category or check box)<br><br>__<br><br>" Not documented	Modified Rankin Scale categories<br>0 – No symptoms at all<br>1 – No significant disability despite symptoms: able to carry out all usual duties and activities.<br>2 – Slight disability: unable to carry out all previous activities, but able to look after own affairs without assistance.<br>3 – Moderate disability: requiring some help, but able to walk without assistance.<br>4 – Moderate to severe disability: unable to walk without assistance, and unable to attend to own bodily needs without assistance.<br>5 – Severe disability: bedridden, incontinent, and requiring constant nursing care and attention.<br>6 – Death.<br>If Item 12.3= Code 20 Expired/dead and the patient expired during hospitalization, item 12.6 can be skipped.	All
12.7	Core<br><SmkCesYN>	Smoking cessation counseling (Check/Not Check)<br>" Yes → Skip to 12.8 if checked	For patients who have smoked at least one cigarette within the past year, code to indicate that patient received counseling to stop smoking or smoking cessation advice during the hospitalization as documented in progress notes or physician orders at discharge or admissions.<br><br>(Referenced source for "past year": JCAHO website)	All
12.7b	OPTION	Smoking cessation counseling (Check only one)<br>" No<br>" Non-Smoker<br>" Unknown	Check "No" box to indicate that patient who smoked at least one cigarette within the past year did NOT receive counseling to stop smoking or smoking cessation advice during the hospitalization.<br>Check "Non-Smoker" box to indicate that the patient's medical record documents that the patient has not smoked within the past year.<br>Check "Unknown" box to indicate that the patient's smoking history is not documented.	

(continued)

Item	Status<br>**<Variable name>**	Item and Categories	Suggestions, Notes, Definitions, Formats	Stroke Type
12.8	Core<br><LipProfD><br><LipProfR><br><LipProfN>	Date of first lipid profile measurement<br>[Date]<br>“ No lab results<br>“ Not documented	mm/dd/yyyy, Check “Not documented” box to indicate that date is not documented, Check “No lab results” to indicate that the lab results are not available.	Ischem only
12.9	Core<br><LipTotal><br><LipHDL><br><LipLDL><br><LipTri>	Lipid Profile (Enter all measurements)<br>Total Cholesterol |__|__|__| mg/dl<br>HDL |__|__|__| mg/dl<br>LDL |__|__|__| mg/dl<br>Triglycerides |__|__|__| mg/dl		Ischem only
12.10	OPTION<br><LipAdYN>	Was patient on lipid altering drug at admission?<br>(Check only one)<br>“ Yes<br>“ No/Not documented	See Appendix I for list of lipid altering drugs.	Ischem only
12.11	Core<br><LipMedYN>	Was patient on lipid altering drug at discharge?<br>(Check/Not Check)<br>“ Yes	See Appendix I for list of lipid altering drugs.<br>If Item 12.3= Code 20 Expired/dead and the patient expired during hospitalization, item 12.11 can be skipped.	Ischem only
12.12	OPTION<br><DMAdYN>	Was patient on diabetes medication at admission?<br>(Check only one)<br>“ Yes<br>“ No/Not documented	See Appendix J for list of diabetes drugs.	All
12.13	Core<br><DMMedYN>	Was patient on diabetes medication at discharge?<br>(Check/Not Check)<br>“ Yes	See Appendix J for list of diabetes drugs.<br>If Item 12.3= Code 20 Expired/dead and the patient expired during hospitalization, item 12.13 can be skipped.	All
12.14	OPTION<br><HemA1C>	Hemoglobin A1C level |__|__|. |__|%		All
12.15	OPTION<br><HBPAdYN>	Was patient on antihypertensive medication at admission?<br>(Check only one)<br>“ Yes<br>“ No/Not documented	See Appendix K for list of antihypertensive drugs.	All
12.16	OPTION<br><HBPMedYN>	Was patient on antihypertensive medication at discharge?<br>(Check only one)<br>“ Yes<br>“ No/Not documented → Skip to 12.18	See Appendix K for list of antihypertensive drugs.<br>If Item 12.3= Code 20 Expired/dead and the patient expired during hospitalization, item 12.16 can be skipped.	All

(continued)

Item	Status<br><Variable name>	Item and Categories	Suggestions, Notes, Definitions, Formats	Stroke Type
12.17	Core<br><HBPType1><br><HBPType2><br><HBPType3><br><HBPType4><br><HBPType5>	Type of antihypertensive medication (Check all that apply)<br>“ ACE Inhibitors<br>“ Beta Blockers<br>“ Ca++ Channel blockers<br>“ Diuretics<br>“ Other antihypertensives	Reference the document prepared by the Ohio group and distributed previously for classification of individual medications in to these categories.<br>If Item 12.3= Code 20 Expired/dead and the patient expired during hospitalization, item 12.17 can be skipped.	All
12.18	OPTION<br><LastSyst><br><LastDias>	Last blood pressures recorded in the chart prior to discharge<br>Systolic BP |__|__|__| mmHg<br>Diastolic BP |__|__|__| mmHg	If Item 12.3= Code 20 Expired/dead and the patient expired during hospitalization, item 12.18 can be skipped.	All
12.19	OPTION<br><br><AthAd1><br><AthAd2><br><AthAd3><br><AthAd4><br><AthAd5><br><AthAd6><br><AthAd7>	Was patient on anti-thrombotic medications on admission? (Check all that apply)<br>“ Aspirin<br>“ Aggrenox<br>“ Warfarin/Coumadin<br>“ Ticlopidine/Ticlid<br>“ Dipyridamole/Persantine<br>“ Clopidogrel/Plavix<br>“ Not Given	See Appendix H for list of synonyms/inclusions of anti-thrombotic drugs.	Ischem only
12.20	Core<br><br><AthDsc01><br><AthDsc02><br><AthDsc03><br><AthDsc04><br><AthDsc05><br><AthDsc06><br><AthDsc07><br><AthDsc08><br><AthDsc09><br><AthDsc10><br><AthDsc11>	Was patient on anti-thrombotic medications on discharge? (Check all that apply)<br>“ Aspirin<br>“ Aggrenox<br>“ Warfarin/Coumadin<br>“ Ticlopidine/Ticlid<br>“ Dipyridamole/Persantine<br>“ Clopidogrel/Plavix<br>“ Heparin SQ<br>“ Heparin IV<br>“ LMW Heparin<br>“ Other anti-thrombotic<br>“ Not Given	See Appendix H for list of synonyms/inclusions of anti-thrombotic drugs.<br>If Item 12.3= Code 20 Expired/dead and the patient expired during hospitalization, item 12.20 can be skipped.	Ischem only

(continued)

Item	Status <Variable name>	Item and Categories	Suggestions, Notes, Definitions, Formats	Stroke Type
12.21	Core	If anti-thrombotic medications were not given (Item 12.20), indicate reasons for non-treatment at discharge.<br>(Check all that apply)	See Appendix H for list of synonyms/inclusions of anti-thrombotic drugs.<br>If Item 12.3= Code 20 Expired/dead and the patient expired during hospitalization, item 12.21 can be skipped.	Ischem only
	<NoAThr01>	" Transferred to another facility		
	<NoAThr02>	" Peptic ulcer (current)		
	<NoAThr03>	" Intracranial surgery/biopsy (current)		
	<NoAThr04>	" Refused Tx		
	<NoAThr05>	" Terminal/comfort care on day of arrival or during the stay		
	<NoAThr06>	" Unrepaired intracranial aneurysm (hx or current)		
	<NoAThr07>	" Expired prior to discharge		
	<NoAThr08>	" Terminal illness (life expectant less than 6 months)		
	<NoAThr09>	" Aortic dissection (current)		
	<NoAThr10>	" Discharged against medical advice		
	<NoAThr11>	" CVA, hemorrhagic (hx or current)		
	<NoAThr12>	" Planned surgery with 7 days following discharge		
	<NoAThr13>	" Allergy to or complication r/t aspirin, ticlopidine, clopidogrel, dipyridamole and warfarin (hx or current)		
	<NoAThr14>	" Brain/CNS cancer (hx or current)		
	<NoAThr15>	" Antithrombotics (Aggrenox, aspirin, dipyridamole, clopidogrel, ticlopidine) considered but not prescribed		
	<NoAThr16>	" Bleeding disorder		
	<NoAThr17>	" Extensive/metastatic cancer (hx or current)		
	<NoAThr18>	" Risk of bleeding (current)		
	<NoAThr19>	" Hemorrhage, any type (hx or current)		
	<NoAThr20>	" Unknown		

(continued)

Item	Status<br><Variable name>	Item and Categories	Suggestions, Notes, Definitions, Formats	Stroke Type
12.22	Core<br><br><br><AFibAT01><br><AFibAT02><br><AFibAT03><br><AFibAT04><br><AFibAT05><br><AFibAT06><br><AFibAT07><br><AFibAT08><br><AFibAT09><br><AFibAT10>	If atrial fibrillation documented (item 10.1 = yes) and patient not taking coumadin/warfarin/heparin/heparinoids, indicate reasons for non-treatment (Check all that apply)<br>“ Risk for bleeding<br>“ Risk for falls<br>“ Mental status<br>“ Liver disease<br>“ Terminal illness<br>“ Patient refused, reason not specified<br>“ Patient refused, did not want risk<br>“ Discontinued due to bleeding<br>“ On ASA as a regular medication<br>“ Arthritis requiring NSAIDs or ASA	See Appendix L for list of synonyms to reasons for non-treatment variables.<br>If Item 12.3= Code 20 Expired/dead and the patient expired during hospitalization, item 12.22 can be skipped.	Ischem only
13	OPTION <Notes>	**Abstractor Notes**	Open text field for any abstractor comments.	

* Final version dated April 3rd, 2002
