# Supplementary material for: Inter-rater reliability of data elements from a prototype of the Paul Coverdell National Acute Stroke Registry
Source: BMC Neurol. 2008 Jun 11;8:19. doi: 10.1186/1471-2377-8-19 (PMC2442121; doi:10.1186/1471-2377-8-19)
Supplement: Additional file 2 — Data Elements For Paul Coverdell National Acute Stroke Registry. Full listing of current data elements for the PCNASR as defined in 2007. Includes Item number, variable name, coding, definitions and formats. [file 1471-2377-8-19-S2.pdf]

Data Elements For Paul Coverdell National Acute Stroke Registry

This table contains both core data elements and optional data elements that are identified under the column labeled “Status”. Core elements are data elements that must be collected by States funded to conduct the Paul Coverdell National Acute Stroke Registry. Optional elements can be added to State registries. If States choose to collect data on an optional element, the definition and response categories in the table should be used. This table may be subject to minor revisions after consultation with funded States.

Item	Status<br><Variable name>	Text Prompt	Field Type	Legal Values	Suggestions, Notes, Definitions, Formats
**1**		**Demographic Data**			
**1.1**	Core<br><Age>	Age |__|__|__| years	Numeric<br>### = 3-digit		Age on the day of admission. Calculated from date of birth in medical record.
**1.2**	Core<br><Gender>	Gender (Check only one)	Numeric<br># = 1-digit	1 - Male<br>2 - Female	Determined by the ER admissions document or the intake/face sheet/hospital admissions database.
**1.3**	Core<br><RaceW><br><RaceAA><br><RaceAs><br><RaceHPI><br><br><RaceAIAN><br><RaceOth><br><RaceOthS><br><RaceUnk>	*Race (Check all that apply)*<br>White<br>Black or African American<br>Asian<br>Native Hawaiian or Other Pacific Islander<br>American Indian or Alaskan Native<br>Other<br>Specify<br>Unknown	 Boolean<br>Boolean<br>Boolean<br>Boolean<br><br>Boolean<br>Boolean<br>Text (25)<br>Boolean		Determined by the ER admissions document or the intake/face sheet/hospital admissions database. If not specified, check “Unknown.”<br><br>A 25 character text field to specify variable <RaceOth> verbatim
**1.4**	Core<br><Hisp>	Ethnicity (Check only one)	Numeric<br># = 1-digit	1 - Hispanic or Latino<br>0 - Not Hispanic or Latino	Determined by the ER admissions document or the intake/face sheet/hospital admissions database.
**1.5**	Core<br><br><HlthInsM><br><HlthInsN><br><HlthIns>	*Health insurance status (Check all that apply)*<br>Medicare<br>No insurance/not documented<br>Other	 <br>Boolean<br>Boolean<br>Boolean		Determined by the ER admissions document or the intake/face sheet/hospital admissions database.
**1.6**	Optional<br><Reside>	Place of Residence (Check only one)	Numeric<br># = 1-digit	1 - Home<br>2 - Nursing Home/Hospice<br>9 -Other/Unknown	Nursing home/hospice definition includes any type of long-term care facility

**2**		**Pre-Hospital/Emergency Medical System (EMS) Data**			
**2.1**	Optional<br><br><InHosp>	Is this an in-patient stroke case?	Numeric<br># = 1-digit	1 - Yes → Skip to section 5 (Imaging)<br>0 - No	In-patient stroke does not meet the Coverdell case definition. If some hospitals collect information regarding inpatient stroke, they should answer this optional question, to enable accurate identification of the Coverdell subset.
**2.2**	Core<br><EMSMode>	Arrival Mode (Check only one)	Numeric<br># = 1-digit	1 – EMS/AIR from scene<br>2 – EMS/AIR – hospital transfer<br>3 – Other<br>9 – Not documented	"Other" includes private transportation (e.g. cab, bus, car, walk-in, etc.).
**2.3**	Core<br><PlaceRcd>	Where was patient received?	Numeric<br># = 1-digit	1 - Emergency Department/Urgent Care<br>2 - Direct Admit<br>9 - Not documented	This question refers to route of patient arrival. Direct admit refers to type of admissions that circumvent ED and might (but not always) include admissions from clinics/urgent care centers and transfers.
**2.4**	Core<br><EMSRecD><br><EMSRecT><br><EMSrcDND><br><EMSrcTND>	<u>*Date & time call received by EMS*</u><br>_ _/ _ _/_ _ _ _<br>_ _:_ _<br>Date Not documented<br>Time Not documented	 Date MMDDYYYY<br>Time HHMM<br>Boolean<br>Boolean		As recorded on the EMS trip sheet or other similar documentation.
**2.5**	Optional<br><EMSGCS><br><EMSGCSND>	Glasgow Coma Scale (GCS)?<br>_ _<br>Not documented	Numeric<br>## = 2-digit<br>Boolean	Range: 3 to 15	Only assess for hemorrhagic stroke patients.

**3**		**Hospital Arrival Data**			
**3.1**	Core<br><br><br><EDTriagD><br><br><EDTriagT><br><br><EDTrgDND><br><EDTrgTND>	Date & time of arrival to this Hospital<br><br>_ _ / _ _ / _ _ _ _<br><br>_ _: _ _<br><br>Date Not documented<br>Time Not documented	 <br><br>Date MMDDYYYY<br><br>Time HHMM<br><br>Boolean<br>Boolean		Documents the earliest time when hospital/ED was aware that there was a patient at their facility that needed to be evaluated.<br><br>Determined by earliest documented time of patient arrival in hospital records, which may be the time of triage or ED check-in, ICU admission (for direct admissions), etc.<br><br>mm/dd/yyyy; 24-hour clock, Check “Not documented” box to indicate that either date or time is not documented.
**4**		**Hospital admission data**			
**4.1**	Core<br><br><HospadD>	Date of hospital admission<br><br>_ _ / _ _ / _ _ _ _	 <br>Date<br>MMDDYYYY		Date of official admission to a hospital’s inpatient service.<br><br>Hospital arrival date and admission date are usually the same for direct admissions but frequently differ for ED admissions.

**4.2**	Core<br><br><PreDxSH><br><PreDxIH><br><PreDxIS><br><PreDxTIA><br><PreDxISU><br><PreDxSNS><br><PreDxHNS><br><br><PreDxNoS>	*Presumptive hospital admission diagnosis at the time of admission related to stroke (check all that apply)*<br><br>Subarachnoid hemorrhage<br>Intracerebral hemorrhage<br>Ischemic stroke<br>Transient ischemic attack<br>Ischemic stroke of uncertain duration<br>Stroke not otherwise specified<br>Hemorrhagic stroke not otherwise specified<br>No stroke related diagnosis	Boolean<br>Boolean<br>Boolean<br>Boolean<br>Boolean<br>Boolean<br>Boolean<br><br>Boolean		This is most likely different from official hospital admission diagnosis and tries to identify presumptive diagnosis at the time of hospital admission. It applies to transfer diagnosis, direct admission diagnosis or ED discharge/hospital admission diagnosis.<br><br>Example: a patient with official diagnosis of “right-sided weakness” might have presumptive diagnosis of stroke in the admission notes. Presumptive diagnosis reflects what diagnosis a patient is evaluated for from the perspective of medical personnel.<br><br>Presumptive diagnosis can often be derived from physicians’ hospital admission, transfer, or ED discharge notes and is often accompanied with keywords such as “suspected”, “presumed”, “probable”, “rule out”, etc.
**4.3**	Core<br><AmbStatA>	Ambulation status prior to the current event.. (Check only one.)	Numeric<br># = 1-digit	1 - Able to ambulate independently w/ or w/o device<br>2 - With Assistance (from person)<br>3 - Unable to Ambulate<br>9 - Not documented	Definition of “ambulate independently": includes assistance with cane, walker, etc., as long as the person can walk without the assistance of another human being. The “current event” is the time of symptoms onset which lead to the current admission.<br>.

**4.4**	Optional <EDDiagCD>	ICD-9 ED discharge/hospital admission diagnosis related to stroke<br>_ _ _. _ _	###.## 5 – digit, 2 decimal places		For admissions through ED, direct admissions or transfers. This is the official diagnosis upon being admitted to a hospital's inpatient service. If ICD-9 ED discharge codes are not assigned by your hospital, enter 000.00; so missing data can be differentiated from data that were not available.<br><br>ICD-9 codes will not be used to collect retrospective data for the Coverdell registry. These codes will only be used to ascertain why these cases were missed prospectively and improve prospective Methodology.

**5**		**Imaging**			
**5.1**	Core<br><br><br><ImageD><br><br><ImageT><br><br><br><ImageSE><br><br><br><ImageDND><br><ImageTND><br><ImageOut>	Date & time of initial brain imaging (not time dictated)<br><br>_ _/ _ _/ _ _ _ _;<br><br>_ _: _ _<br><br><br>Is this a specific or estimated date/time?<br><br><br>Date Not documented<br>Time Not documented<br>Outside brain imaging prior to transfer	Date<br>MMDDYYYY<br><br>Time HHMM<br><br><br>Numeric<br># = 1-digit<br><br><br>Boolean<br>Boolean<br>Boolean	1 - Specific<br>2 - Estimated	This question applies to the initial brain image done at your hospital. Use Outside brain imaging if there were no images done at your hospital and check “Outside brain imaging prior to transfer” box.<br><br>mm/dd/yyyy; 24-hour clock, Check “Not documented” box to indicate that either date or time is not documented. Time is only documented for imaging at the registry hospitals. You do not need to document outside brain imaging time.<br><br>Disregard imaging done at the registry hospital 24 or more hours after the admission/arrival time; these images are usually done to check the progression of the disease and not for initial work-up. If there is no imaging done within the first 24 hours of admission/arrival, either do not record imaging or record outside imaging as appropriate.<br><br>Determined by date/time recorded on the image itself or the date/time that the scan was performed as noted in the dictated report. Time of dictation is not recorded by the abstractor.<br><br>If specific time is not available, calculate estimated time from when the patient left the ED and when the patient returned from the ED.
**5.2**	Core<br><ImageRes>	Initial brain imaging findings?	Numeric<br># = 1-digit	1 – Hemorrhage<br>0 - No hemorrhage<br>9 - Not available	Hemorrhage includes hemorrhagic stroke.

**6**		**Signs and Symptoms Onset**			
**6.1**	Core<br><OnsetD><br><OnsetT>	Date and time of symptom onset<br>_ _/ _ _/ _ _ _ _<br>___: ____ (go to < OnsetRel >)	Date MMDDYYYY<br>Time HHMM		Military (24 hour) time should be used. The purpose of this question is to conservatively identify/estimate time of symptoms onset. Use “when symptoms begin” if time of symptom onset can be reliably derived; otherwise use “last seen normal”. For example, mark “last seen normal” if patient felt fine before going to bed but developed symptoms sometime overnight. If there are multiple conflicting time points in medical records (for either “symptom onset” or “last seen normal”) then use the earliest one.<br><br>If there are more than one instance of onset of symptoms, document the time ultimately responsible for this admission. If initial instance of symptom was resolved completely prior to the second instance that lead to admission, than use time of the second instance. If distinction is not clear, refer to physicians’ notes for any suggestions to which instance they consider beginning of symptoms responsible for this admission. For example: document earliest time if patient developed right arm weakness that evolved into right-sided weakness unless there is a clear indication in physician’s notes that start of symptoms was latter rather than former.<br><br>If there is a specific indirect reference to time that can be used for calculation with a reasonable accuracy, then the abstractor should do so. For example: if time of ED arrival is known and documentation says that “symptoms started 45 minutes prior to
	<OnsetTEs>	Time of day of symptom onset (if a specific time is not provided above):	Numeric<br># = 1-digit	1 - Morning (6:00 am – 11:59 am)<br>2 - Afternoon (noon – 5:59 pm)<br>3 - Evening (6:00 pm – 11:59 pm)<br>4 - Overnight (midnight – 5:59 am)<br>9 - Not documented	
	<OnsetDUN><br><OnsetTUN>	Documented as “Unknown date”<br>Documented as “Unknown time”	Boolean<br>Boolean		
	<OnsetDND><br><OnsetTND>	Date not documented<br>Time not documented	Boolean<br>Boolean		
	<OnsetRel>	Is this date/time when (select one):	Numeric<br># = 1-digit	1 - Patient last known normal<br>2 - When symptoms began	
	Optional<br><onsetcom>	Comments	Text 200		

					the arrival” or “patient was seen without symptoms 1 hour prior to the arrival” then an abstractor should calculate and document the time.<br><br>If neither specific date and/or time nor specific indirect reference to timing (as described above) is provided, then date and/or time should not be left blank.<br><br>Time of day of symptom onset should be filled out if time is not provided and there is a specific reference to a time of day. For example: “patient was last seen normal sometime during the afternoon”<br><br>Documented as “unknown” question should be checked if the respective date and/or time is not available and there is a specific reference in a patient’s records that date and/or time was unknown.<br><br>Use “date and/or time not documented” responses if neither of the respective (date for dates and time for times) previous questions have been answered. Do NOT use “not documented” if “documented as “unknown”” has been checked.<br><br>“Is this date and time when (select one)” should be filled out whenever any information to date and/or time has been entered. It should not be filled out if “not documented” or “documented as “unknown”” have been checked for both date and time.

**6.2**	Optional<br><NIHStrkS><br><br><br><br><NIHStrkN>	First NIH Stroke Scale total score recorded by hospital personnel<br>(enter score <u>or</u> check box)<br>__ __<br><br>Not documented	 <br>Numeric<br>## = 2-digit<br><br>Boolean	Range: 00 to 42	Total score maximum is 2-digit<br>First NIHSS can be recorded by either the MD or a member of the "stroke team" (including a PA or RN).
**7**		**Thrombolytic Treatment**			
**7.1**	Optional<br><TrmMec><br><br><TrmMechD><br><TrmMecT><br><TrmMecDN><br><TrmMecTN>	 Was IA clot removal device used?<br><br>_ _/ _ _/ _ _ _ _ ___: ____<br>Date Not documented<br>Time Not documented	 Boolean<br><br>Date MMDDYYYY<br>Time HHMM<br>Boolean<br>Boolean		This includes IA mechanical therapy that includes; for example: guidewires, microcatheters, balloon angioplasty or snares such as the MERCI retriever. Or similar therapy that is defined at the state level.

**7.2**	Core<br><br><TrmIVT><br><TrmIVTD><br><TrmIVTT><br><TrmIVTDN><br><TrmIVTTN><br><br><TrmIVM><br><TrmIVMD><br><TrmIVMT><br><TrmIVMDN><br><TrmIVMTN><br><br><TrmIAM><br><TrmIAMD><br><TrmIAMT><br><TrmIAMDN><br><TrmIAMTN><br><br><TrmNSM><br><TrmNSMD><br><TrmNSMT><br><TrmNSMDN><br><TrmNSMTN><br><br><TrmN>	Did patient receive thrombolytic therapy? (Check all that apply)<br>IV tPA transferring hospital<br><br>_ _/ _ _/ _ _ _ _ ___: ____<br>Date Not documented<br>Time Not documented<br><br>IV tPA in this hospital<br><br>_ _/ _ _/ _ _ _ _ ___: ____<br>Date Not documented<br>Time Not documented<br><br>IA thrombolytic in this hospital<br><br>_ _/ _ _/ _ _ _ _ ___: ____<br>Date Not documented<br>Time Not documented<br><br>Thrombolytic, not specified<br><br>_ _/ _ _/ _ _ _ _ ___: ____<br>Date Not documented<br>Time Not documented<br><br>No	Boolean<br>Date MMDDYYYY<br>Time HHMM<br>Boolean<br>Boolean<br><br>Boolean<br>Date MMDDYYYY<br>Time HHMM<br>Boolean<br>Boolean<br><br>Boolean<br>Date MMDDYYYY<br>Time HHMM<br>Boolean<br>Boolean<br><br>Boolean<br>Date MMDDYYYY<br>Time HHMM<br>Boolean<br>Boolean<br><br>Boolean		Do not include thrombolytic therapy for indications other than ischemic stroke. That is, do not include intra-cerebral venous infusion for cerebral venous thrombosis, intraventricular infusion for intraventricular hemorrhage, intraparenchymal infusion for percutaneous aspiration of intracerebral hematoma, myocardial infarction, PE, or peripheral clot.<br><br>Principal investigators/clinical consultants from state registries should develop and maintain a list of thrombolytic solutions that data abstractors can use for reference. IA thrombolytic includes only chemical solutions. Mechanical devices such as "Clot retrieval devices" are NOT part of IA thrombolytic treatment.<br><br>Go to section 8 (non-treatment with thrombolytics).
**7.3**	Optional<br><TrmExp>	Was patient on any kind of thrombolytic investigational protocol?	Numeric<br># = 1-digit	1 - Yes<br>9 - No/Not documented	Mark yes if medical records suggest that some kind of investigational thrombolytic protocol was used during provision of care.
**7.4**	Optional<br><ExpType>	Specify	Text 200		Optional field to describe nature of the experimental protocol described in 7.2

**7.5**	Core<br><br><br><br><br><ThrmCmpS><br><ThrmCmpL><br><br><ThrmCmpN>	Complications of thrombolytic therapy<br>(Check all that apply among responses)<br><br>Symptomatic intracranial hemorrhage<br>Life threatening, serious systemic hemorrhage<br>None/not documented	 <br><br><br><br>Boolean<br>Boolean<br><br>Boolean		Definition for symptomatic hemorrhage: CT hemorrhage within 36 hours shows intracranial bleed AND physician’s notes indicate clinical deterioration due to hemorrhage.<br><br>Definition for systemic hemorrhage: ≥ 3 transfused units of blood within 7 days or discharge (which ever is earlier) AND physician note attributing bleeding problem as reason for transfusion.<br><br>Check “None” box to indicate that patient did not experience either symptomatic intracranial hemorrhage or life threatening, serious systemic hemorrhage as complications of thrombolytic therapy.<br><br>If Section 7 is completed → Skip to Section 9 (Medical History).

**8**		**Non-Treatment with Thrombolytics**<br>Section 8 completed only if thrombolytic therapy not given or started.			

**8.1**	Core<br><NonTrtA><br><NonTrtOD><br><NonTrtDR><br><NonTrtTD><br><NonTrtUH><br><NonTrtRI><br><NonTrtCT><br><NonTrtSM><br><NonTrtSS><br><NonTrtSZ><br><NonTrtST><br><NonTrtIC><br><NonTrtFR><br><NonTrtNC><br><NonTrtHH><br><NonTrtAG><br><NonTrtIB><br><NonTrtPC><br><NonTrtPT><br><NonTrtGL><br><NonTrtIV><br><NonTrtIL><br><NonTrTX><br><NonTrtOC><br><NonTrtOt><br><NonTrtND>	If thormbolytics were not given, did a responsible provider specify in the chart one or more of the following reasons for not giving thrombolytics?<br>(Check all that apply.)<br>Delay in patient arrival<br>Delay in CT ordered to CT done<br>Delay in CT done to CT read<br>Other time delay<br>Uncontrolled hypertension<br>Rapid improvement<br>CT findings<br>Stroke severity – Too mild<br>Stroke severity – Too severe<br>Seizure at onset<br>Recent surgery/trauma (<15 days)<br>Recent IC surgery (3 mo.) head trauma/stroke<br>Pt./Family refused<br>Consent not obtainable<br>History of intracranial hemorrhage or brain aneurysm or vascular malformation or brain tumor<br>Age<br>Active internal bleeding (<22 days)<br>Platelet count (<100,000)<br>Abnormal aPTT or PT<br>Glucose < 50 mg/dl or > 400 mg/dl<br>No IV access<br>Life expectancy < 1 year or severe co-morbid illness<br>Investigative therapy for acute ischemic stroke<br>Other: ____________________<br>Not documented	Boolean<br>Text 25 characters<br>Boolean		Intent of this question is to capture documented or inferred contraindication. Check item if documented by physician or nurse in admission or discharge notes. Do not document evidence from outside physician or nurse notes without some kind of inference that it played a factor in the decision-making process for not giving thrombolytics.<br><br>Variable <NonTrtOt> is a text field to record Other responses.

**9**		**Medical History**			
**9.1**	Core<br><br><br><MedHisST><br><MedHisCS><br><MedHisMI><br><br><MedHisPA><br><MedHisAF><br><MedHisHT><br><MedHisDL><br><MedHisDM><br><MedHisSM><br><br><br><MedHisVP>	Documented past medical history of any of the following:<br>(Check all that apply.)<br>Stroke/Transient ischemic attack/VBI<br>Carotid stenosis.<br>Myocardial infarction (MI) or Coronaryartery disease (CAD)<br>Peripheral arterial disease<br>Atrial fibrillation<br>Hypertension<br>Dyslipidemia<br>Diabetes mellitus (DM)<br>Smoking - patient has smoked at leastone cigarette within the past year<br>Valve prosthesis	 <br><br>Boolean<br>Boolean<br>Boolean<br><br>Boolean<br>Boolean<br>Boolean<br>Boolean<br>Boolean<br>Boolean<br><br><br>Boolean		Check item if documented by physician or nurse in admission or discharge notes.<br><br>Coronary Artery Disease (CAD) - Documented evidence in medical record of a specific mention of the presence of CAD or a revascularization procedure, such as coronary bypass grafting or stenting. Documentation of angina is NOT enough to be defined as CAD.<br><br>Smoking history – patient has smoked at least one cigarette within the past year.
**10**		**In-Hospital Diagnostic Procedures and Treatment**			
**10.1**	Optional<br><NeuroYN>	Was a neurologist, neurosurgeon, or a stroke team involved in patient's care during hospitalization?	Numeric<br># 1-digit	1 - Yes<br>9 - No/Not documented	This can be obtained by viewing SOAP or consult notes in a patient's record.<br><br>[Note: This question should not have a default value.]
**10.2**	Core<br><br><AFibYN>	Was atrial fibrillation present in the hospital?	Numeric<br># 1-digit	1 - Yes<br>9 - No/Not documented	Atrial fibrillation includes A-flutter, and paroxysmal atrial fibrillation.<br>If this is the sole criteria for whether a patient should receive anticoagulation therapy then the definition should include a reference to PAF as these patients also should be treated with anticoagulation<br><br>[Note: This question should not have a default value. This response is used as a filter in item 12.14]

**10.3**	Core<br><br><AThrTime>	Time of initiation of any anti-thrombotic therapy after arrival. (Check only one)	Numeric<br># 1-digit	1 - 0 – 24 hours<br>2 - 24 – 48 hours<br>3 - >48 hours<br>4 - Not initiated<br>9 - Not documented	This question applies to all anti-platelet and anticoagulant agents.<br><br>Documented in the patient's record and calculated from the time of patient's arrival
**10.4**	Core<br><DVTAmbul>	Was patient ambulating without assistance within 48 hours after arrival?	Numeric<br># 1-digit	1 - Yes – Skip to question 10.6<br>0 - No<br>9 - Not documented	Can be found in physicians' or nurses' notes. Definition of "ambulate independently": includes assistance with cane, walker, etc., as long as the person can walk without the assistance of another human being. Independent ambulation should not only be happening under "controlled" circumstances, such as with physical therapist that visits patient for a very limited time. There should be documentation that patient was ambulating more than 50 feet independently, or independently out of bed to bathroom without supervision.
**10.5**	Core<br><DVTProYN>	Was DVT prophylaxis initiated within 48 hours after arrival?	Numeric<br># 1-digit	1 - Yes<br>0 - No<br>9 - Not documented	Determination if medication and/or devices were ordered and initiated within 48 hours of hospital admission for prophylaxis against the formation of deep venous thrombosis. Inclusion:<br><br>1) Low-dose, sub-Q, subcutaneous, unfractionated ("regular") heparin, Low Molecular Weight (LMW) heparin (enoxaparin, dalteparin, nadroparin, danaparoid, hirudin, bivalirudin, heparinoids) or trial based antithrombin agent or other agent not listed above<br><br>2) Intravenous heparin, IV heparin.

					3) Pneumatic Compression Stockings, compression socks, Intermittent compression devices, ICDs, (TED Hose do NOT apply)<br><br>4) Warfarin, Aldocumar, Anisindione, Anisinidine, Athrombin, Athrombin-K, Barr Warfarin Sodium, Barr's Warfarin Sodium, Carfin, Coufarin, Coumadan, SodicoCoumadin, Coumadina, Coumadine, Dicumarol, Dicoumarol, Indandione, Liquamar, Marevam, MarevanMiradon, Orfarin, Panwarfin, Panwarfarin, Phenprocoumon, Sefarin, Sofarin, Uniwarfin, Waran, Warfarin, Warfarin Sod, Warfarin Sodium, Warfilone Sodium, Warifilone<br><br>**Select:**<br><br>Yes = if any of these medications or treatments are ordered for the patient and initiated even if "DVT Prophylaxis" as the indication is not specifically documented in the order or progress notes. Therapeutic anticoagulation also meets the criteria for prophylaxis. Also, select “Yes” if a patient continues receiving one of the DVT prophylaxis listed above that was started prior to admission.<br><br>No = if none of the above methods are ordered and initiated for the patient.<br><br>Example: Patient BMC025 is admitted to the in-patient unit following treatment with thrombolytic therapy. Thirty-six hours after administration of rt-PA, the patient is

					not able to ambulate and requires two people to assist him. His medications do not include any anticoagulants and he is on Plavix. Data Entry will be to select No. If patient had TED hose, data entry would also be to select "No”.
**10.6**	Core<br><DysphaYN>	Screening for dysphagia prior to any oral intake?	Numeric<br># 1-digit	1 - Yes<br>0 - No<br>2 – NPO<br>9 - Not documented	Documented in nurse’s notes or speech/swallowing consult. Types of dysphagia screen include simple water swallow test, formal speech evaluation, and None of these are screens but diagnostic tests! The screen was done prior to any of these being performed<br><br>Answer NPO only if patient was kept NPO during their entire hospital stay and discharged/Deceased/transferred NPO. This response should not be used in any other circumstances. Data abstractors should wait until either patient is taken off NPO or discharged prior to answering this question.
**11**		**Other In-Hospital Complications**			
**11.1**	Optional<br><DVTDocYN>	Was DVT documented?	Numeric<br># 1-digit	1 - Yes<br>0 - No	Confirmed by ultrasound or venous imaging. [JCAHO defines this as objectively confirmed DVT based on duplex ultrasound, contrast venography, CT with contrast or CT venogram, MR imaging or MR venography]<br>Items 11.1 and 11.2 refer to in-hospital acquired events requiring treatment. Pre-existing conditions and therapy present prior to admission should not be counted in responding to this data element.

**11.2**	Optional<br><PneumYN>	Was pneumonia documented?	Numeric<br># 1-digit	1 - Yes<br>0 – No<br>.	Physician documentation of definite or possible. This only applies to nosocomial pneumonia that occurs after 48 hours of admission. The question excludes pneumonias that were present prior to admission or started within 48 hours of admission.<br><br>Items 11.1 and 11.2 refer to in-hospital acquired events requiring treatment. Pre-existing conditions and therapy present prior to admission should not be counted in responding to this data element.
**12**		**Discharge Data**			
**12.1**	Core<br><DschrgD><br><DschrgND>	Date of discharge from hospital<br>_ _/ _ _/_ _ _ _<br><br>Not documented	Date MMDDYYYY<br><br>Boolean		mm/dd/yyyy, Check “Not documented” box to indicate that date is not documented.<br><br>Indicate the date the patient was discharged from acute care, left against medical advice, or expired during this stay.
**12.2**	Core<br><br><ICD9StDx><br><br><ICD9StND>	ICD-9 discharge diagnosis related to stroke<br>__ __ __ . __ __<br><br>Not present	###.## 5 – digit, 2 decimal places<br><br>Boolean		Use XXX.YY format; allow only one ICD-9 code.<br><br>Determined by ICD-9 code recorded in chart.
**12.3**	Core<br><ICD9PrDx>	What is principal discharge ICD-9 diagnosis?<br>__ __ __ . __ _	###.## 5 – digit, 2 decimal places		This is the diagnosis at the time of discharge.

**12.4**	Core	Final hospital diagnosis related to stroke that was ultimately responsible for this admission (check all that apply)			This is the final admission diagnosis after completion of all diagnostic procedures, examinations and consultations.<br><br>Note, this may be different from the presumptive hospital admission diagnosis and the final discharge diagnosis.
	<AdmDxSH>	Subarachnoid hemorrhage	Boolean		
	<AdmDxIH>	Intracerebral hemorrhage	Boolean		
	<AdmDxIS>	Ischemic stroke	Boolean		
	<AdmDxTIA>	Transient ischemic attack	Boolean		
	<AdmDxISU>	Ischemic stroke of uncertain duration	Boolean		
	<AdmDxSNS>	Stroke not otherwise specified	Boolean		
	<AdmDxHNS>	Hemorrhagic stroke not otherwise specified	Boolean		
	<AdmDxNoS>	No stroke related diagnosis	Boolean		

**12.5**	Core<br><DschDest>	Discharge destination (Check only one.)	Numeric<br>## 2-digit	01 - Discharged to home care or self care (routine discharge)<br>02 - Discharged/transferred to another short term general hospital for inpatient care<br>03 - Discharged/transferred to a skilled nursing facility (SNF) with Medicare certification.<br>04 - Discharged/transferred to an intermediate care facility<br>05 - Discharged/transferred to another type of institution for inpatient care<br>06 - Discharged/transferred to home under care of organized home health service organization<br>07 - Left against medical advice or discontinued care<br>08 - Discharged/transferred to home under care of a home IV drug therapy provider<br>20 - Expired (or did not recover-Christian Science Patient).<br>41 - Expired in medical facility, such as hospital, SNF, ICF or freestanding hospice (Hospice).<br>50 - Hospice - home<br>51 - Hospice - medical facility<br>61 - Discharged/transferred within this institution to hospital-based Medicare approved swing bed<br>62 - Discharged/transferred to an inpatient rehabilitation facility (IRF) including rehabilitation distinct part units of a hospital<br>63 - Discharged/transferred to a Medicare certified long term care hospital (LTCH)<br>64 - Discharged/transferred to a nursing facility certified under Medicaid but not certified under Medicare	[Reference source: UB-92 Codes]<br><br>Code numbers correspond to UB-92 codes for discharge. Use only the documented UB-92 codes; do not infer from a given information..<br><br>Data can be found in:<br>1) Administrative Data: UB-92, Field Location: 22<br>2) Medical Record: Discharge instruction sheet, discharge summary, face sheet, nursing discharge notes, physician orders, social service note, transfer record
**12.6**	Core<br><AmbStatD>	Ambulation status at discharge. (Check only one.)	Numeric<br># 1-digit	1 - Able to ambulate independently w/ or w/o device<br>2 - With Assistance (from person)<br>3 - Unable to Ambulate<br>9 - Not documented	Definition of “ambulate independently": includes assistance with cane, walker, etc., as long as the person can walk without the assistance of another human being.<br>If Item 12.5= Code 20 Expired/dead and the patient expired during hospitalization, item 12.6 can be skipped.

**12.7**	Core<br><SmkCesYN>	Smoking cessation counseling	Numeric<br># 1-digit	1 - Yes<br>9 - No/Not documented	For patients who have smoked at least one cigarette within the past year (JCAHO), code to indicate that patient received counseling to stop smoking or smoking cessation advice during the hospitalization as documented in progress notes or physician orders at discharge or admissions. It does not meet criteria of "Yes" to simply advise the patient that smoking is bad for their health.<br><br>Smoking cessation therapies such as patch, gum, etc, are also equivalent to counseling.
**12.8**	Core<br><br><LipTotal><br><LipHDL><br><LipLDL><br><LipTri>	Lipid Profile (Enter all measurements)<br>Total Cholesterol |__|__|__| mg/dl<br>HDL |__|__|__| mg/dl<br>LDL |__|__|__| mg/dl<br>Triglycerides |__|__|__|__| mg/dl	Numeric ### 3-digit<br>Numeric ### 3-digit<br>Numeric ### 3-digit<br>Numeric #### 4-digit		Determined from lab results in a patient's hospital record.<br><br>If there is more than one measurement, select the latter one.<br><br>Also, answer this question if there is a lipid profile taken within the preceding 30 days that is available in the medical records. Actual values must be available in the medical records for this question to be answered.
**12.9**	Core<br><LipAdmYN>	Was patient on lipid altering drug at admission?<br>(Check only one)	Numeric # 1-digit	1 - Yes<br>9 - No/Not documented	Can be obtained from patient's medical history. The intent of the question is to identify patients with a documented history of hyperlipidemia that is being therapeutically treated.

**12.10**	Core<br><LipDisYN>	Was patient on lipid altering drug at discharge?	Numeric # 1-digit	1 - Yes<br>9 - No/Not documented	If Item 12.5= Code 20 Expired/dead and the patient expired during hospitalization, item 12.10 can be skipped.<br>Questions 12.10 and 12.11 are intended to record whether patient was prescribed these medication on discharge (was it part of the discharge medications list?). We do not currently check to see whether the prescription was filled.
**12.11**	Core<br><br><HBPTreat>	Was patient on antihypertensive medication at discharge?	Numeric # 1-digit	1 - Yes<br>9 - No/Not documented	If Item 12.5= Code 20 Expired/dead and the patient expired during hospitalization, item 12.11 can be skipped.<br>Questions 12.10 and 12.11 are intended to record whether patient was prescribed these medication on discharge (was it part of the discharge medications list?). We do not currently check to see whether the prescription was filled.
**12.12**	Core<br><br><br><AthDscAs><br><AthDscAc><br><AthDscWC><br><AthDscTT><br><AthDscCP><br><AthDscIV><br><AthDscLM><br><AthDscOT><br><AthDscND>	Was patient on anti-thrombotic medications on discharge?<br>(Check all that apply)<br>Aspirin<br>Aggrenox<br>Warfarin/Coumadin<br>Ticlopidine/Ticlid<br>Clopidogrel/Plavix<br>Unfractionated heparin IV<br>Full dose LMW heparin<br>Other anti-thrombotic<br>Not given/ND	 <br><br>Boolean<br>Boolean<br>Boolean<br>Boolean<br>Boolean<br>Boolean<br>Boolean<br>Boolean<br>Boolean		If Item 12.5= Code 20 Expired/dead and the patient expired during hospitalization, item 12.12 can be skipped.

**12.13**	Core	If anti-thrombotic medications were not given (Item 12.12), indicate reasons for non-treatment at discharge. (Check all that apply)			If Item 12.5= Code 20 Expired/dead and the patient expired during hospitalization, item 12.13 can be skipped.<br><br>The intent of this question is to document contraindications to anti-thrombotic therapy. While the abstractors may make reasonable inferences from available doctors' notes, they should not actively search in the patient's record for contraindications.
	<NoAThrTR>	Transferred to another facility	Boolean		
	<NoAThrPU>	Peptic ulcer (current)	Boolean		
	<NoAThrIS>	Intracranial surgery/biopsy (current)	Boolean		
	<NoAThrRT>	Refused Tx	Boolean		
	<NoAThrTC>	Terminal/comfort care on day of arrival or during the stay	Boolean		
	<NoAThrUA>	Unrepaired intracranial aneurysm (hx or current)	Boolean		
	<NoAThrEX>	Expired prior to discharge	Boolean		
	<NoAThr6M>	Terminal illness (life expectant less than 6 months)	Boolean		
	<NoAThrAD>	Aortic dissection (current)	Boolean		
	<NoAThrAA>	Discharged against medical advice	Boolean		
	<NoAThrCV>	CVA, hemorrhagic (hx or current)	Boolean		
	<NoAThr7S>	Planned surgery with 7 days following discharge	Boolean		
	<NoAThrAL>	Allergy to or complication r/t aspirin, ticlopidine, clopidogrel, dipyridamole and warfarin (hx or current)	Boolean		
	<NoAThBC>	Brain/CNS cancer (hx or current)	Boolean		
	<NoAThrNP>	Antithrombotics (Aggrenox, aspirin, dipyridamole, clopidogrel, ticlopidine) considered but not prescribed	Boolean		
	<NoAThrBD>	Bleeding disorder	Boolean		
	<NoAThrMC>	Extensive/metastatic cancer (hx or current)	Boolean		
	<NoAThrRB>	Risk of bleeding (current)	Boolean		
	<NoAThrHM>	Hemorrhage, any type (hx or current)	Boolean		
	<NoAThrOT>	Other	Boolean		
	<NoAThND>	Not Documented	Boolean		

**12.14**	Core<br><br><br><br><br><br><AFibATRB><br><AFibATRF><br><AFibATMS><br><AFibATLD><br><AFibATTI><br><AFibATRN><br><AFibATRR><br><AFibATDB><br><AFibATAS><br><AFibATAR><br><AFibATOT><br><AFibATND>	If atrial fibrillation documented (item 10.2 = yes) and patient not taking coumadin/warfarin/heparin/ Heparinoids upon discharge, indicate reasons for non-treatment (Check all that apply)<br>Risk for bleeding<br>Risk for falls<br>Mental status<br>Liver disease<br>Terminal illness<br>Patient refused, reason not specified<br>Patient refused, did not want risk<br>Discontinued due to bleeding<br>On ASA as a regular medication<br>Arthritis requiring NSAIDs or ASA<br>Other<br>Not documented	 <br><br><br><br><br>Boolean<br>Boolean<br>Boolean<br>Boolean<br>Boolean<br>Boolean<br>Boolean<br>Boolean<br>Boolean<br>Boolean<br>Boolean<br>Boolean		If Item 12.5= Code 20 Expired/dead and the patient expired during hospitalization, item 12.14 can be skipped.<br><br>If treatment was refused, please select “Patient refused, reason not specified” or “Patient refused, did not want risk” even if the family refused rather than the patient.

**12.15**	Core<br><br><EducRes>	Was there documentation that patient and/or caregiver received education and/or resource materials.	Numeric # 1-digit	1 - Yes<br>2 – No<br>3 – Not documented	Percent of patients and/or caregivers receiving stroke education including but not limited to education regarding diet and alcohol counseling, and associated co-morbid conditions including hypertension, diabetes and hyperlipidemia upon discharge.<br><br>Suggested Data sources: Medical record – flow sheets, clinician encounter notes, teaching sheets, consult notes (e.g., social work consult), care plans.<br><br>Hints to abstractors: Record documentation must reflect that the patient and/or caregiver was received education and/or resource materials. If the organization uses standardized written materials that contain the required components, i.e., etiology, risk factor modification, social service resources, then documentation of receipt of these tools is adequate.<br><br>If Item 12.5= Code 20 Expired/dead and the patient expired during hospitalization, item 12.15 can be skipped.

**12.16**	Core<br><br><RehaPlan>	Is there documentation in the record that the patient was assessed for or received rehabilitation services.	Numeric # 1-digit	1 - Yes<br>2 – No<br>9 – Not documented	Answer "Yes" only if there is evidence that specific plans for rehabilitation were made, or if there is a reason for not needing rehabilitation and it is documented in the chart. A note stating “rehabilitation should be considered” does not qualify as “Yes” answer.<br><br>Suggested Data sources: Physician orders, progress notes, consultant reports, referral forms, clinical logs, multidisciplinary progress notes.<br><br>Examples of rehabilitation team members include: Physiatrist, Neuro-psychologist, Occupational Therapist, Physical Therapist, Nursing, Speech Therapist, Other
**12.17**	Optional	<u>*If answered “Yes” question 12,16, check all services that apply*</u>			
	<Rehcons>	Consult for rehabilitation services	Boolean		
	<Rehasses>	Assessment/treatment by members of the rehabilitation team	Boolean		
	<Rehrecei>	Patient received rehabilitation services during hospitalization	Boolean		
	<Rehtrans>	Patient transferred to rehabilitation facility	Boolean		
	<Rehrefer>	Patient referred to rehabilitation services following discharge	Boolean		
	<Rehineli>	Patient ineligible to receive rehabilitation services (e.g., symptoms resolved, poor prognosis, patient unable to tolerate rehabilitation therapeutic regimen)	Boolean		
	<Rehrefus>	Patient/family refused rehabilitation services	Boolean		
