# Supplementary material for: Inter-rater reliability of data elements from a prototype of the Paul Coverdell National Acute Stroke Registry
Source: BMC Neurol. 2008 Jun 11;8:19. doi: 10.1186/1471-2377-8-19 (PMC2442121; doi:10.1186/1471-2377-8-19)
Supplement: Additional file 3 — Inter-rate reliability for selected data elements from the Paul Coverdell National Acute Stroke Registry. Inter-rater reliability results for all of the PCNASR data elements. Includes Item number, variable name, sample size, Prevalence index (PI), lower confidence interval of Kappa statistic, and Bias index (BI). Results were not generated for date or text fields, or for those variables that had insufficient sample size or when the PI was extreme (> 0.90 or < -0.90). [file 1471-2377-8-19-S3.pdf]

Online Only Table. *Inter-rate reliability for selected data elements from the Paul Coverdell National Acute Stroke Registry.*

**Item***		***N***	**PI**	**Kappa (LCL)**	**BI**
**Coverdell Stroke Sub-type**		104			
	❝ Ischemic Stroke		0.24	0.61 (0.46)	-0.07
	❝ Intracranial hemorrhage (ICH)		-0.86	0.93 (0.79)	0.01
	❝ Subarachnoid hemorrhage (SAH)		-0.89	0.90 (0.72)	0.01
	❝ Stroke of uncertain type		-0.92	—†	0.06
	❝ TIA		-0.64	0.70 (0.52)	-0.01
	❝ Hemorrhagic Stroke of uncertain type		-1.00	—†	—†
	❝ Ischemic Stroke of uncertain duration		-0.95	—†	0.03
**Demographic Data**					
1.2	Gender (Check only one): ❝ Female (positive); ❝ Male (negative); ❝ Unknown (negative)	104	0.16	0.98 (0.94)	0.01
1.3	Race (Check all that apply)	104			
	❝ White		0.40	0.55 (0.38)	0.13
	❝ Black or African American		-0.66	0.97 (0.90)	0.01
	❝ Asian or Pacific Islander		-1.00	—†	—†
	❝ American Indian or Alaskan Native		-1.00	—†	—†
	❝ Other		-0.98	—†	0.00
	❝ Not documented		-0.76	0.07 (-0.13)	-0.11
1.4	Hispanic or Latino origin (Check only one): ❝ Yes (positive); ❝ No/Not documented (negative)	104	-0.97	—†	0.01
1.5	Place of Residence (Check only one): ❝ Nursing home (positive); ❝ Other (negative)	104	-0.88	0.82 (0.58)	-0.02
1.7	Health insurance status (Check all that apply)	104			
	❝ Medicare		0.43	0.83 (0.72)	0.03
	❝ Medicaid		-0.86	0.50 (0.18)	-0.03
	❝ Blue Cross/Blue Shield		-0.11	0.75 (0.62)	0.07
	❝ HMO/PPO		-0.71	0.77 (0.59)	-0.02
	❝ Other private/commercial		-0.72	0.40 (0.15)	-0.03

Item*		*N*	PI	Kappa (LCL)	BI
	“ Other, Specify		-0.93	—†	0.03
	“ Self Pay		-0.89	0.52 (0.15)	0.01
	“ Not documented		-0.94	—†	-0.02
**Pre-Hospital/Emergency Medical System (EMS) Data**					
2.1	Did the stroke meeting the case definition occur in-hospital? (Check only one): “ Yes (positive) → Skip to Section 5 (Imaging); “ No (negative)	104	-0.89	0.90 (0.72)	0.01
2.2	Arrival Mode (Check only one)	104			
	“ Ambulance		-0.40	0.82 (0.69)	0.00
	“ Air		-0.99	—†	-0.01
	“ Ambulance hospital transfer		-0.74	0.70 (0.50)	0.03
	“ Other		-0.14	0.59 (0.43)	-0.01
	“ Not documented		-0.72	0.40 (0.15)	-0.01
2.2b	Was the patient a direct admit? (Check only one): “ Yes (positive); “ No (negative)	98	-0.88	0.47 (0.17)	0.04
**Arrival at Emergency Department (ED) Data**					
3.1	Where did stroke occur? (Check only one)	104			
	“ Work		-0.95	—†	0.01
	“ Home		0.39	0.24 (0.06)	-0.20
	“ Other		-0.80	0.53 (0.26)	-0.05
**Emergency Department (ED)**					
4.1	Date & time of arrival in ED	90			
	[Date/Time]				
	“ Not documented		0.63	0.59 (0.40)	0.07
4.2	Date & time first seen by emergency department medical doctor	90			
	[Date/Time]				
	“ Not documented		0.21	0.44 (0.26)	0.04

Item*		N	PI	Kappa (LCL)	BI
4.3	Evidence in record of consultation or discussion with acute stroke team? (Check only one): “ Yes (positive); “ No (negative) → Skip to Item 4.5		-0.12	0.30 (0.10)	0.01
4.4	If yes, date & time of acute stroke consultation	40			
	[Date/Time]				
	“ Not documented		0.20	0.02 (-0.20)	0.40
4.5	Was stroke or stroke-like symptoms documented in the emergency department evaluation? (Check only one): “ Yes (positive); “ No (negative)	90	0.92	—†	-0.01
4.6	Was stroke/TIA one of the documented ED diagnoses? (Check only one): “ Yes (positive); “ No (negative)		0.86	0.26 (-0.08)	0.03
**Imaging**					
5.1	Type of initial brain image (Check only one.)	104			
	“ CT		0.89	0.71 (0.41)	-0.03
	“ MRI		-0.92	—†	0.04
	“ Not done		-0.99	—†	0.01
	“ Not documented		-0.99	—†	-0.01
5.2	Date & time of initial brain imaging (not time dictated)				
	[Date/Time] → If date & time are documented, skip to Item 5.5				
	“ Not documented → complete Items 5.3 & 5.4		-0.08	0.32 (0.15)	0.17
	“ Outside brain imaging prior to transfer → skip to Item 5.5		-0.88	0.59 (0.26)	0.01
5.3	Date & time patient left the ED to radiology for initial image.	38			
	[Date/Time]				
	“ Not documented		-0.16	0.68 (0.44)	0.00
5.4	Date & time patient returned from radiology following initial imaging to the ED or returned to other location (e.g., med/surg floor, angiography suite--whichever is earlier).	38			

Item*		*N*	PI	Kappa (LCL)	BI
	[Date/Time]				
	“ Not documented		-0.26	0.55 (0.28)	-0.05
5.5	Date & time of earliest documentation of imaging results known to the treating physician.	103			
	[Date/Time]				
	“ Not documented		-0.46	0.25 (0.10)	0.29
5.6	Evidence of intracranial hemorrhage on initial image? (Check only one) “ Yes (positive), “ No (negative), “ Not documented (negative)	103	-0.71	0.84 (0.70)	0.00
**Signs and Symptoms Onset**					
6.1	Is a specific date and time of onset of acute stroke S/S documented or is an estimated time accurate to within a six-hour window available? (Check only one)				
	“ Specific time → skip to 6.2.a / 6.2.b		-0.40	0.51 (0.33)	0.13
	“ Estimated time → skip to 6.3.c / 6.3.d		-0.17	0.22 (0.06)	-0.25
	“ No specified time → skip to 6.4.e		-0.44	0.48 (0.30)	0.12
6.2	IF YES – SPECIFIC TIME:				
	**6.2.a** Specific date and time of onset:				
	[Date/Time]				
	**6.2.b** What was the source of this information?	38			
	“ Witnessed		-0.37	0.21 (-0.05)	0.26
	“ Patient self-report		-0.45	0.06 (-0.11)	0.39
	“ Not documented		-0.26	0.00	-0.74
6.3	IF YES – ESTIMATED TIME:	30			
	Estimated date of onset: [Date]				
	Estimated time of day of onset:				
	“ Morning (6am-11:59am)		-0.23	0.52 (0.23)	0.17
	“ Afternoon (noon-5:59pm)		-0.70	0.61 (0.21)	0.03
	“ Evening (6pm-11:59pm)		-0.70	0.62 (0.23)	0.10
	“ Overnight (midnight-5:59am)		-0.63	0.67 (0.32)	-0.03

Item*		*N*	PI	Kappa (LCL)	BI
**Non-Treatment with Thrombolytics**					
8.1	Physician documented reasons indicated for non-treatment with thrombolytics (these are not necessarily contraindications) (Check all that apply)	86			
	“ Time		-0.83	0.78 (0.54)	0.03
	“ Uncontrolled hypertension		-1.00	—†	0.00
	“ Rapid improvement		-0.97	—†	-0.01
	“ CT findings		-0.95	—†	0.02
	“ Stroke severity – Too mild		-0.91	—†	0.07
	“ Stroke severity – Too severe		-0.99	—†	0.01
	“ Seizure at onset		-1.00	—†	0.00
	“ Recent surgery/trauma (<15 days)		-1.00	—†	0.00
	“ Recent IC surgery (3 mo.) head trauma/stroke		-0.99	—†	0.01
	“ Pt./Family refused		-1.00	—†	0.00
	“ Consent not obtainable		-1.00	—†	0.00
	“ History of intracranial hemorrhage or brain aneurysm or vascular malformation or brain tumor		-0.98	—†	0.00
	“ Age		-0.98	—†	0.02
	“ Active internal bleeding (<22 days)		-1.00	—†	0.00
	“ Platelet count (<100,000)		-1.00	—†	0.00
	“ Abnormal aPTT or PT		-1.00	—†	0.00
	“ Glucose < 50 mg/dl or > 400 mg/dl		-1.00	—†	0.00
	“ No IV access		-1.00	—†	0.00
	“ Life expectancy < 1 year or severe co-morbid illness		-1.00	—†	0.00
	“ Other		-0.94	—†	0.01
	“ Not Documented		0.59	0.54 (0.33)	-0.10
**Medical History**					
9.1	Documented past medical history of any of the following (Check all that apply):	104			
	“ Stroke/Transient ischemic attack/VBI		-0.23	0.59 (0.44)	-0.04

Item*		N	PI	Kappa (LCL)	BI
	“ Myocardial infarction (MI)		-0.71	0.77 (0.59)	0.02
	“ Coronary artery disease (CAD)		-0.26	0.61 (0.45)	-0.05
	“ Atrial fibrillation		-0.64	0.70 (0.52)	0.01
	“ Heart failure (CHF)		-0.65	0.80 (0.65)	-0.04
	“ Valve prosthesis		-0.97	—†	-0.01
	“ Hypertension		0.38	0.73 (0.59)	0.04
	“ Dyslipidemia		-0.40	0.72 (0.58)	0.04
	“ Diabetes mellitus (DM)		-0.48	0.80 (0.67)	0.04
	“ Smoking		-0.59	0.62 (0.43)	0.03
**In-Hospital Diagnostic Procedures and Treatment**					
10.1	Was atrial fibrillation present in the hospital (Check only one): “ Yes (positive); “ No (negative) [Note: This response is used as a filter in item 12.22]		-0.66	0.63 (0.43)	0.07
10.2	Which of the following tests were documented to evaluate the cerebrovasculature? (Check all that apply):				
	“ Duplex ultrasound		0.13	0.75 (0.62)	-0.05
	“ MR Angiogram		-0.41	0.56 (0.39)	0.07
	“ CT Angiogram		-0.97	—†	-0.01
	“ Cerebral Angiogram		-0.83	0.64 (0.37)	-0.02
	“ Transcranial Doppler		-0.89	0.33 (-0.05)	0.01
	“ None documented		-0.54	0.68 (0.51)	0.04
10.4	Time of initiation of any anti-thrombotic therapy after admission. (Check only one)	104			
	“ 0 – 24 hours		0.53	0.55 (0.37)	0.09
	“ >24 hours		-0.90	—†	0.02
	“ Not initiated		-0.67	0.79 (0.63)	-0.06
	“ Not documented		-0.96	—†	-0.05
10.5	If initiated, what anti-thrombotic therapy given during acute hospital care? (Check all that apply):	90			
	“ Aspirin		0.06	0.58 (0.41)	-0.06
	“ Aggrenox		-0.76	0.69 (0.46)	0.00

Item*		*N*	PI	Kappa (LCL)	BI
	“ Warfarin/Coumadin		-0.62	0.64 (0.43)	-0.02
	“ Ticlopidine/Ticlid		-1.00	—†	0.00
	“ Dipyridamole/Persantine		-0.96	—†	-0.04
	“ Clopidogrel/Plavix		-0.28	0.59 (0.42)	0.06
	“ Heparin SQ		-0.69	0.43 (0.19)	0.13
	“ Heparin IV		-0.40	0.74 (0.58)	0.02
	“ LMW Heparin		-0.90	—†	0.03
	“ Other anti-thrombotic		-1.00	—†	0.00
	“ Not Given		-1.00	—†	0.00
10.6	Was DVT prophylaxis initiated by 2nd hospital day (Check only one):	104			
	“ Yes		-0.49	0.43 (0.24)	0.11
	“ No		-0.74	0.54 (0.30)	-0.07
	“ Not applicable (patient ambulating or already receiving anticoagulant)		0.22	0.50 (0.33)	-0.05
	“ Not documented		-0.99	—†	0.01
10.7	Screening for dysphagia (Check only one): “ Yes (Positive); “ No (Negative)	104	-0.32	0.64 (0.48)	0.05
10.8	Was a neurologist or neurosurgeon involved in the care of the patient during hospitalization (Check only one): “ Yes (Positive); “ No (Negative)	104	0.56	0.67 (0.49)	-0.04
10.9	Is there an order in the medical record that limits the medical care provided, such as “Do not resuscitate ” or “Comfort/supportive care only” (Check only one): “ Yes (Positive); “ No (Negative)	104	-0.61	0.67 (0.49)	0.09
**Other In-Hospital Complications**					
11.1	Was DVT documented? (Check only one): “ Yes (Positive); “ No (Negative)	104	-0.95	—†	0.01
11.2	Clinical mention of pneumonia, and treatment with antibiotic for that problem (Check only one): “ Yes (Positive); “ No (Negative)	104	-1.00	—†	0.00

Item*		*N*	PI	Kappa (LCL)	BI
11.3	Clinical mention of urinary tract infection (UTI), and treatment with antibiotic for that problem (Check only one): ❝ Yes (Positive); ❝ No (Negative)	104	-1.00	—†	0.00
**Discharge Data**					
12.1	Date of discharge from hospital	104			
	[Date]				
	❝ Not documented		-0.96	—†	0.04
12.3	Discharge destination (Check only one):	104			
	❝ Code 1. Discharge to home or self-care (routine discharge)		-0.22	0.38 (0.23)	0.26
	❝ Code 2. Discharge/transfer to another short-term general hospital for inpatient care		-0.98	—†	0.02
	❝ Code 3. Discharge/transfer to skilled nursing facility (SNF)		-0.88	0.00	0.13
	❝ Code 4. Discharge/transfer to intermediate care facility (ICF)		-1.00	—†	0.00
	❝ Code 5. Discharge/transfer to another type of institution for inpatient care or referred for outpatient services to another institution		-0.78	0.30 (0.07)	0.14
	❝ Code 6. Discharge/transfer to home under care of organized home health service organization		-0.94	—†	0.06
	❝ Code 7. Left against medical advice or discontinued service		-1.00	—†	0.00
	❝ Code 8. Discharge/transfer to home under care of a home IV provider		-1.00	—†	0.00
	❝ Code 9. Admitted as inpatient to hospital		-1.00	—†	0.00
	❝ Code 20. Expired/dead		-0.95	—†	0.05
	❝ Other UB-92 codes		-1.00	—†	0.00
	❝ Disposition not documented		-1.00	—†	0.00
12.5	Functional status at discharge (Check only one):	104			
	❝ Able to ambulate independently		0.19	0.52 (0.36)	0.04

Item*		*N*	PI	Kappa (LCL)	BI
	“ Ambulates with assistance from another individual		-0.64	0.19 (-0.03)	-0.09
	“ Not able to ambulate		-0.71	0.69 (0.49)	0.02
	“ Not documented		-0.84	0.43 (0.12)	0.03
12.6	Modified Rankin Scale at discharge (Enter scale category or check box)	104			
	[Score]				
	“ Not documented		-0.95	—†	-0.01
12.8	Date of first lipid profile measurement				
	[Date]				
	“ No lab results		0.06	0.69 (0.55)	-0.06
	“ Not documented		-0.80	0.42 (0.14)	0.03
12.11	Was patient on lipid altering drug at discharge? (Check/Not Check): “ Yes		-0.35	0.78 (0.65)	0.04
12.13	Was patient on diabetes medication at discharge (Check/Not Check): “ Yes		-0.55	0.86 (0.75)	0.03
12.16	Was patient on antihypertensive medication at discharge (Check only one): “ Yes (positive); “ No/Not documented (negative) → Skip to 12.18		0.25	0.60 (0.44)	0.12
12.17	Type of antihypertensive medication (Check all that apply):	55			
	“ ACE Inhibitors		-0.29	0.21 (-0.01)	0.24
	“ Beta Blockers		-0.16	0.48 (0.24)	0.00
	“ Ca++ Channel blockers		-0.42	0.65 (0.43)	0.04
	“ Diuretics		-0.44	0.78 (0.59)	-0.02
	“ Other antihypertensives		-0.18	0.21 (-0.05)	-0.05
12.20	Was patient on anti-thrombotic medications on discharge (Check all that apply):	104			
	“ Aspirin		-0.17	0.76 (0.64)	-0.02
	“ Aggrenox		-0.83	0.76 (0.52)	-0.02
	“ Warfarin/Coumadin		-0.58	0.94 (0.86)	0.00
	“ Ticlopidine/Ticlid		-1.00	—†	0.00
	“ Dipyridamole/Persantine		-0.99	—†	-0.01

Item*	N	PI	Kappa (LCL)	BI
“ Clopidogrel/Plavix		-0.38	0.75 (0.62)	0.05
“ Heparin SQ		-0.91	—†	0.03
“ Heparin IV		-0.98	—†	0.00
“ LMW Heparin		-0.94	—†	0.00
“ Other anti-thrombotic		-1.00	—†	0.00
“ Not Given		-0.60	0.76 (0.60)	-0.02

*Items not reported due to type of variable or insufficient sample size: 1.1, 1.6, 2.3, 2.4, 2.4b, 2.5, 2.6, 2.7, 2.8, 2.8b, 2.9, 2.10, 2.11, 6.4, 6.5, 6.6, 6.7, 7.1, 7.2, 7.3, 7.4, 7.5, 8.2, 10.3, 12.2, 12.2a, 12.4, 12.7, 12.7b, 12.9, 12.10, 12.12, 12.14, 12.15, 12.18, 12.19, 12.21, 12.22, 13. †Insufficient information or non-calculable. PI = Prevalence Index. BI = Bias Index. LCL = Lower 95% Confidence Limit.
